# Synthesis, Characterisation, and Antimicrobial Evaluation of *Spirostachys africana* Extract-Based Silver Nanoparticles Against Selected Bacterial Pathogens, Including Antibiotic-Resistant Strains

**DOI:** 10.3390/nano16201317

**Published:** 2026-10-18

**Authors:** Murunwa Felicia Nemaguvhuni, Amidou Samie

**Affiliations:** Department of Biochemistry and Microbiology, Faculty of Science, Engineering and Agriculture, University of Venda, Thohoyandou 0950, South Africa; 17022786@mvula.univen.ac.za

**Keywords:** respiratory tract infections, *Spirostachys africana*, plant-mediated synthesis, silver nanomaterials, antibacterial efficacy, microdilution assay, nanoparticle characterization, transmission electron microscopy

## Abstract

The rise of antibiotic-resistant (ABR) bacterial pathogens is a major global health concern, especially in the case of Respiratory tract infections (RTIs), which can make treatment extremely difficult. This study explores the green synthesis, characterization, and antimicrobial activity of silver nanoparticles from the medicinal plant *Spirostachys africana* extracts (SA-AgNPs) using methanol, ethanol, acetone, and water solvents. Optimized synthesis was achieved with 5 mM silver nitrate and incubation at 4 °C, producing stable nanoparticles. UV-Vis analysis revealed surface plasmon resonance peaks at 480 nm, confirming nanoparticle formation. X-ray diffraction indicated face-centered cubic crystalline structures with sizes ranging from 9 to 19 nm. Transmission electron microscopy revealed predominantly spherical nanoparticles with core sizes between 5 and 136 nm. Antimicrobial assays demonstrated greater activity of the SA-AgNPs than the corresponding crude extracts against selected bacterial pathogens, including strains with documented antibiotic-resistance phenotypes. Notably, acetone-extract conjugated nanoparticles produced the largest inhibition zone of 22 mm against *Pseudomonas aeruginosa*. Bark methanol, ethanol, and acetone conjugated nanoparticles were highly effective against *Acinetobacter baumannii*, with minimum inhibitory concentrations as low as 0.08 mg/mL, while the methanol leaf extract showed activity against *P. aeruginosa* (0.09 mg/mL). This is the first study to synthesize and characterize nanoparticles from *S. africana*, showing that AgNPs synthesized from the plant offer promising, eco-friendly alternatives for combating antibiotic-resistant respiratory pathogens. Further in vivo studies and mechanistic investigations are recommended to advance their therapeutic development.

## 1. Introduction

Antibiotic resistance (ABR) among bacterial pathogens is a critical global health crisis, causing an estimated 4.95 million deaths worldwide in 2019, including 1.27 million directly attributable to resistant infections [1]. This escalating burden complicates the treatment of common infections such as respiratory tract infections (RTIs). RTIs affect both the upper respiratory tract (e.g., rhinitis, sinusitis, pharyngitis, tracheitis) and the lower respiratory tract (e.g., bronchitis, pneumonia) and are primarily transmitted via respiratory droplets [2,3]. Common resistant bacterial pathogens associated with RTIs include Carbapenem-resistant (CR) *Acinetobacter baumannii*, CR *Pseudomonas aeruginosa*, CR *Escherichia coli*, and Methicillin-resistant *Staphylococcus aureus* (MRSA), many of which are listed as priority pathogens by the World Health Organization (WHO), highlighting the urgent need for novel therapeutic strategies [4].

Nanoparticles (NPs), are defined as tiny particles composed of various materials and shapes, typically measuring between 1 and 100 nm in size [5]. Among these, metal nanoparticles (MNPs) such as silver nanoparticles (AgNPs) have emerged as promising antimicrobial agents due to their distinctive physicochemical properties and multiple modes of action against microorganisms [5,6,7]. AgNPs exhibit potent antimicrobial activity at low concentrations with minimal toxicity, making them particularly attractive in addressing ABR [8]. Their antimicrobial efficacy is influenced by factors such as particle size, shape, concentration, and surface coating, which have been extensively studied [9]. Additionally, AgNPs have versatile applications beyond medicine, including drug delivery, catalysis, sensors, cosmetics, and environmental remediation [10,11].

Various methods exist for the synthesis of AgNPs, including chemical, physical, and biological approaches. Chemical methods, although widely used, often involve toxic reagents, while physical techniques tend to be expensive and require harsh conditions like high temperatures or pressures [12]. In contrast, biological methods, also known as green synthesis, have gained attention as safe, simple, rapid, eco-friendly, and cost-effective alternatives. Green synthesis employs biological agents such as microorganisms and plants to reduce metal ions and stabilize nanoparticles. Plant-mediated green synthesis is particularly advantageous due to the abundance, diversity, and ease of handling of phytochemicals that act as both reducing and capping agents [13].

Medicinal plants have long been used traditionally to treat various infections, including RTIs, through preparations such as infusions, decoctions, steam inhalations, and topical applications [14]. These plants are rich in bioactive phytochemicals, including coumarins, terpenoids, glycosides, flavonoids, phenols, polyphenols, vitamins, and steroids, which contribute to their therapeutic efficacy and facilitate NPs biosynthesis [15,16]. Plant extract-based NP have demonstrated enhanced bioactivities with applications in antimicrobial, anticancer, anti-inflammatory treatments, gene therapy, drug delivery, and bioimaging [17].

The medicinal plant *Spirostachys africana* (*S. africana*), also known as “*Tamboti*,” is traditionally employed for treating a range of ailments including dysentery, diarrhea, colds, flu, fever, headaches, nose or gum bleeding, and respiratory infections [18,19,20,21]. Common preparations include teas (infusions or decoctions), steam inhalations for respiratory conditions, and topical applications for wounds and infections [20,22]. Phytochemical analyses reveal that the bark and leaves of *S. africana* contain alkaloids, phenols, flavonoids, coumarins, saponins, steroids, terpenoids, and tannins, compounds known for antimicrobial, anti-inflammatory, antioxidant, and antimalarial activities [20,21]. The bark and roots are boiled into infusions or soft porridges for treating respiratory illnesses and other conditions such as stomach pains, colds, and fevers [18]. Studies have demonstrated *S. africana* extracts’ antimicrobial activities against pathogens including *Klebsiella pneumoniae*, *Staphylococcus aureus*, *Escherichia coli*, *Salmonella typhi*, *Shigella dysenteriae*, and *Vibrio cholerae* [20,21,23].

Despite the ethnomedicinal importance of *S. africana*, its potential for the green synthesis of AgNPs has not been extensively explored. Given the urgent need for alternative antibacterial agents against priority bacterial pathogens, this study aimed to synthesize and characterize AgNPs using *S. africana* bark and leaf extracts prepared with different solvents and to evaluate their antibacterial activity against selected bacterial pathogens. The synthesized AgNPs were characterized using UV-Vis spectroscopy, X-ray diffraction (XRD), transmission electron microscopy (TEM), and Fourier-transform infrared spectroscopy (FTIR), while their antibacterial activity was evaluated using agar diffusion, broth microdilution, and minimum bactericidal concentration (MBC) assays. The synthesized AgNPs exhibited characteristic surface plasmon resonance (SPR) bands, crystalline structures, and nanoscale morphologies, with selected formulations demonstrating notable antibacterial activity, particularly against *P. aeruginosa* and *S. aureus*.

## 2. Materials and Methods

### 2.1. Plant Material Collection and Preparation

Ethical clearance for the study was obtained from the University of Venda ethics committee. The ethical clearance reference number was FSEA/22/BMY/07. The plant material, including stem bark and leaves of *S. Africana*, was harvested in one of the villages in the Nzhelele region, Limpopo province, South Africa. The plant material was collected on the 23rd of June 2023 from a single tree from a population of about 100 individuals in the plains of Ha-Matsa Village, with the GPS coordinates: 22.8715 S, 29.9657 E.

The plant material was identified by the Department of Botany, University of Venda, and the Voucher No. SAF002/22 was given for the original samples collected. Tap water was used to clean the plant material to eliminate the soil residues. The material was then cut into smaller pieces and dried for two weeks at room temperature. The plant material was further ground into a fine powder using an electric grinder model FZ102 (Tianjin Taisite Instruments, Tianjin, China) and stored in a sealed glass jar in the dark at ambient temperature for further use.

**Figure 1:**
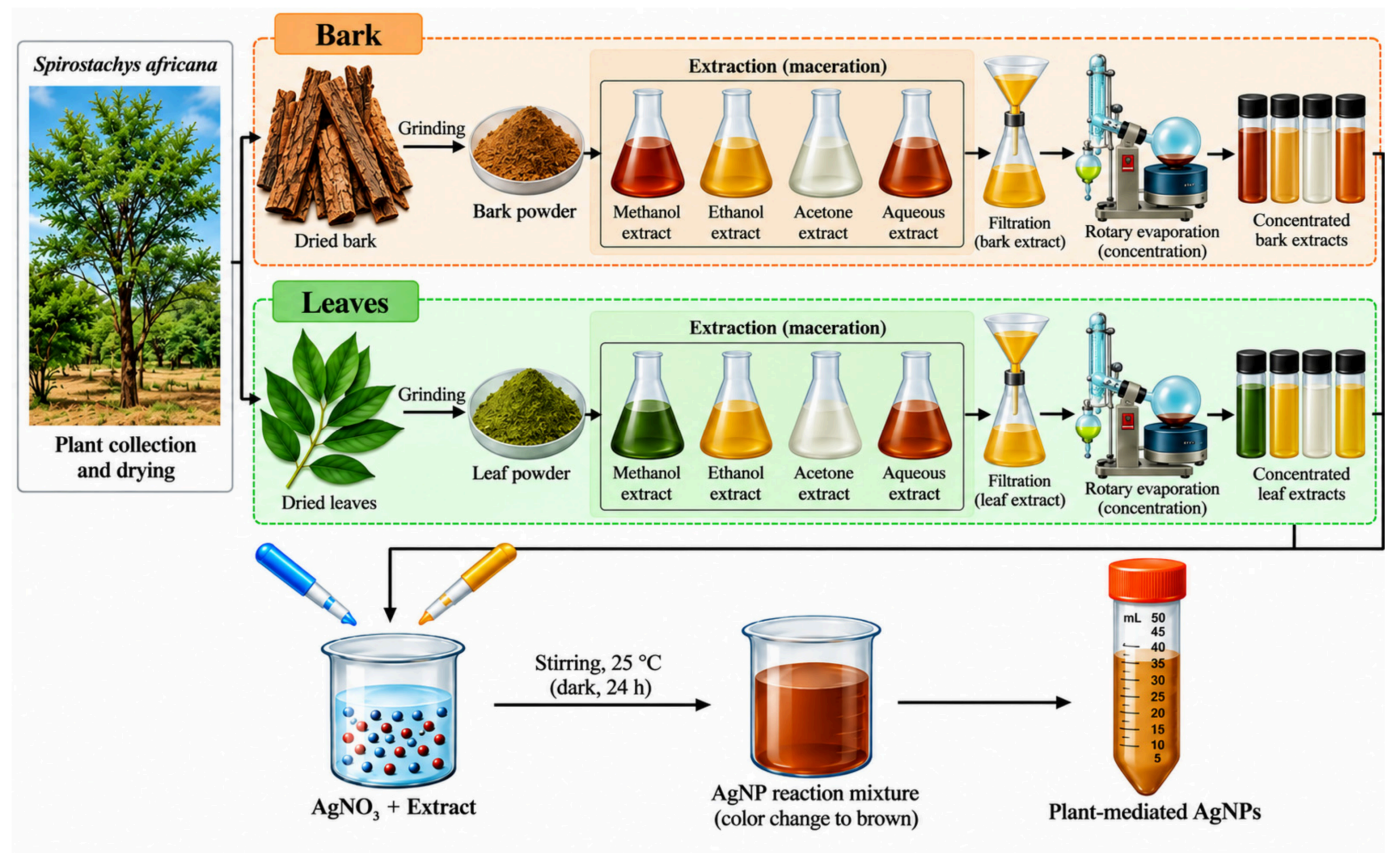
*S. africana* plant collection, extraction, and green synthesis of SA-AgNPs.

Solvents, including methanol, ethanol, acetone (Rochelle Chemical, Johannesburg, South Africa), and distilled water were used for the extraction. Briefly, fifty grams of the ground plant material (stem bark or leaves) were separately soaked in 500 mL of each solvent for 72 h, with frequent shaking. The crude extracts were filtered and dried using a rotary evaporator (Buchi Rotavapor R-300, Cole-Parmer, Vernon Hills, IL, USA) under pressure at 40 °C [24]. The resulting organic extract concentrates were further allowed to air-dry in a fume hood and subsequently reconstituted in 10% (*v*/*v*) dimethyl sulfoxide (DMSO) to obtain the required concentrations for AgNP synthesis (Figure 1). For water extracts, the filtrate was first frozen at −80 °C and then placed in a freeze dryer (EPIC Freeze Dryer, Millrock Technology, Kinston, NY, USA) for 24 h. All the plant extracts were stored at 4 °C for future use.

### 2.2. Green Synthesis of AgNPs Mediated by S. africana Extracts

The synthesis of silver nanoparticles (SA-AgNPs) was conducted following the methods outlined by Liaqat et al. [25], with slight modifications. In a concise procedure, 10 mL from each *S. africana* extract (10 mg/mL), prepared by dissolving the dried extract powder in 10% (*v*/*v*) dimethyl sulfoxide (DMSO) aqueous solution, was introduced separately dropwise into a 250 mL sterile glass beaker containing 40 mL of silver nitrate (1, 2 and 5 mM) solution at the ratio (1:4 *v*/*v*) at room temperature, with shaking. The reaction mixture was prepared in the dark to avoid photoactivation of silver nitrate (AgNO_3_). Each sample was labeled, covered with aluminum foil, and incubated in the dark for 24 h. After that, AgNPs were stored at different temperatures (at 4 and 25 °C) to study the effect of temperature. The study also explored the impact of reaction time on SA-AgNP production at different time intervals (1 h, 24 h, seven days, month 2, and month 3). The reduction of Ag^+^ to Ag^0^ was primarily monitored by visual observation of color change from light yellow to blackish brown or yellowish brown and from dark yellow to dark brown or reddish brown, and their UV-Vis spectra were continuously assessed to determine their stability state.

### 2.3. Characterization of S. africana Crude Extracts and Its Green Synthesized AgNPs

#### 2.3.1. Ultraviolet-Visible Spectrophotometry Analysis

The formation of SA-AgNPs was further validated by measuring the absorbance of the reaction mixture using Ultraviolet-visible spectrophotometry (UV-Vis) [26,27]. Briefly, the SA-AgNPs samples were diluted to a 1:24 ratio with the same solvent. Dilutions were performed in 2 mL centrifuge tubes, and then 200 μL of the diluent was transferred into a 96-well plate in triplicates. The plate containing the NP samples underwent scanning in a wavelength range from 200 to 800 nm using a SpectraMax M3 spectrophotometer (Molecular Devices, San Jose CA, USA) at a resolution of 2 nm. This procedure was done for all the NP samples except for the acetone extract-based NPs, where 2 mL of the diluted solution was dispersed in a glass cuvette to read for absorbance, as acetone solvent was not suitable for the 96-well plate. Absorption peaks in the UV-Vis spectra confirming the production of NPs were observed.

#### 2.3.2. X-Ray Diffraction Unit (XRD) Analysis

The synthesized SA-AgNPs were filtered using a Munktell filter paper 3HW (Lasec, Cape Town, South Africa), and the pallets were analyzed by an XRD (Pan Analytical, X-pert pro, Netherland). The XRD measurements of SA-AgNPs were conducted using a Cu-Kα radiation source (λ = 1.5406 Ǻ) in the scattering range m (2θ) of 20–80 on an instrument operating at a voltage of 45 kV and a current of 40 mA. The XRD spectroscopy was employed to ascertain the synthesized NPs’ crystalline nature, phase variety, and grain size. The Debye-Scherrer formula was applied to calculate the average crystalline size:

$$D=\frac{0.9\lambda }{\beta cos\;\theta }$$

where: D represents the crystallite size (measured in nm), 0.9 represents the Scherrer constant (*k*), *λ* denotes the wavelength of the X-ray source, *θ* signifies the peak position (measured in radians), and *β* represents the full width at half maximum (FWHM) [28].

#### 2.3.3. Transmission Electron Microscopy

Transmission electron microscopy (TEM) with excellent resolution and magnification was employed to study the prepared SA-AgNPs, as it allows direct visualization of the particles and provides detailed information about their size, shape, and distribution [29,30]. The TEM analysis was conducted using an FEI Tecnai-12 electron microscope (FEI, Eindhoven, The Netherlands) operated at an acceleration voltage of 120 kV. The samples were prepared by immersing a copper grid in the NP solution and quickly removing it to enable drying, forming a thin film. Obtained images from TEM were analyzed to determine particle sizes using ImageJ software (Version 1.54s) by measuring the diameter (nm) of each particle. Following this, the acquired values were plotted using OriginPro 2023 software and subjected to statistical analysis to determine both the average size and standard deviation. Error was computed based on the obtained values. Notably, in this analysis, 100 NPs from distinct regions were examined for each sample.

#### 2.3.4. Fourier-Transform Infrared Spectroscopy (FTIR) Analysis

The functional groups that were responsible for biological activities present in *S. africana* extracts and SA-AgNPs were identified by FTIR using the Bruker ALPHA FT-IR Spectrophotometer (Bruker Scientific Private Limited, Mumbai, India). The analysis was done by placing 500 μL of the plant extracts or NP samples on top of the FTIR spectrophotometer chamber. Percentage transmittance measurements were recorded within the wavelength range of 400–4000 cm^−1^, as outlined by Patel et al. [31].

### 2.4. Antimicrobial Activity of S. africana Extracts and Nanoparticles

#### 2.4.1. Preparation of Microorganisms

For the evaluation of the antimicrobial activities of the extracts and nanoparticles, four respiratory bacterial pathogens listed by the WHO as critical priority pathogens of antibiotic resistance were used. These included *Acinetobacter baumannii* (ATCC BAA-1605), *Pseudomonas aeruginosa* (ATCC 27853), *Escherichia coli* (ATCC BAA-2469), and *Staphylococcus aureus* (NCTC 12493). The bacterial strains used in this study were purchased from Anatech Instruments (Gauteng, South Africa). The bacterial suspensions were then adjusted to 0.5 McFarland standard (Sigma, St. Louis, MO, USA) (1.5 × 10^8^ CFU/mL) before antimicrobial testing.

#### 2.4.2. Agar Well-Diffusion Method

The antibacterial activities of *S. africana* extracts and SA-AgNPs were determined using the agar well-diffusion method, as previously described by Bhakya et al. [32]. Initially, Mueller-Hinton (MH) agar medium (Separations, Johannesburg, South Africa) plates were prepared to culture the bacterial strains. A 200 μL volume of the test organisms with turbidity adjusted to 0.5 McFarland standard was inoculated over the surface of MH agar using a sterile spreader and allowed to dry for 30 min. Six evenly spaced wells with a diameter of 8 mm were aseptically created by gently punching the agar with a tip. Each well was filled with 50 μL of each plant extract (10 mg/mL) and SA-AgNPs (2.68 mg/mL). The plates were left for 1 h to allow diffusion of the treatment samples into the inoculated agar. Finally, the agar plates were incubated at 37 °C for 24 h. Antibiotic lomefloxacin hydrochloride (10 mg/mL) (Sigma-Aldrich, Darmstadt, Germany) was used as a positive control, while 10% dimethyl sulfoxide (DMSO) (Rochelle Chemical, Johannesburg, South Africa) served as the negative control. To assess the efficacy of the experiment, the diameter of the clear zone, indicating the inhibition of bacterial growth, was meticulously measured in millimeters using a ruler. For the precision and reliability of the results, the experiment was repeated three times for each test organism. The growth inhibition zones were subsequently recorded and presented in graphs. This experiment was also conducted for three months to investigate the effect of time and stability of the SA-AgNPs.

#### 2.4.3. Determination of the Minimum Inhibitory Concentrations (MIC)

The minimum inhibitory concentrations (MICs) of the plant extracts and SA-AgNPs against the priority pathogens (*A. baumannii*, *P. aeruginosa*, *E. coli*, and *S. aureus*) were determined using the microdilution assay as previously described [33]. A 10% DMSO solution was used as a negative control, while lomefloxacin hydrochloride at 10 mg/mL was used as a positive control. Stability testing was also conducted using the microdilution assay over three months. The trials were labeled Experiment 1, Experiment 2, and Experiment 3 for the first, second, and third months, respectively. This was done in triplicate for each experiment.

#### 2.4.4. Determination of Minimum Bactericidal Concentration (MBC)

For minimum bactericidal concentration (MBC), approximately 5 µL of each sample was taken from the wells with no observable bacterial growth during the MIC tests and subcultured onto Mueller-Hinton agar plates and subsequently incubated at 37 °C for 24 h. The MBC was defined as the minimum concentration at which no bacterial growth was observed on the agar. The assay was performed in triplicate (*n* = 3) [34].

### 2.5. Statistical Analysis

Data were recorded in Microsoft Excel, and the biological experiments conducted in triplicate (n = 3) were expressed as the mean ± standard error (SEM). One-way analysis of variance (ANOVA) was applied to assess the significance of individual differences at a *p* < 0.05 level. Significant means were compared with Tukey’s test via GraphPad Prism 8 software. Graphs were plotted using Excel and OriginPro 2023b software.

## 3. Results

### 3.1. Synthesis and Stability Evaluation of S. africana-AgNPs

The results indicated that the extracts from the *S. africana* plant effectively reduced AgNO_3_ ions to AgNPs within 24 h. This was evidenced by the change from yellow to blackish brown/yellowish-brown and from brown to dark brown/reddish-brown (Figure S1). These color changes were associated with excitation of the surface plasmon resonance (SPR) of the formed AgNPs and were supported by the UV-Vis spectra, which showed absorption bands within 397–480 nm.

The stability of SA-AgNPs was investigated by optimization of various parameters such as reaction times, AgNO_3_ concentrations, and temperatures. Initially, the color intensity increased with reaction time, showing similar trends in *S. africana* methanol bark/leaf extract conjugated AgNPs (SABM-AG/SALM-AG) and *S. africana* ethanol bark/leaf extract conjugated AgNPs (SABE-AG/SALE-AG) samples, transitioning from light brown to blackish black or light yellow to yellowish brown (Figure S2). *S. africana* acetone bark/leaf extract conjugated AgNPs (SABA-AG/SALA-AG) samples changed from light yellow to dark brown/yellowish brown. Meanwhile, *S. africana* aqueous bark/leaf extract-conjugated AgNPs (SABD-AG/SALM-AG) samples exhibited varying trends from dark yellow to dark brown over time (Figures S2 and S3).

Increasing AgNO_3_ concentration from 1 to 5 mM altered NP color intensity, shifting from light yellow/brown at 1 mM to darker shades at 2 and 5 mM, accompanied by a shift in the SPR peak towards higher wavelengths. The study found that higher concentrations of AgNO_3_ led to a quicker color change. Furthermore, it was observed that SA-AgNPs incubated at 4 °C reacted more slowly than those at 25 °C, with color intensity increasing more over time at room temperature than in the fridge. These observations were consistent with the corresponding UV-Vis spectral changes, indicating that reaction temperature influenced the rate of AgNP formation.

### 3.2. Characterization of SA-AgNPs

#### 3.2.1. Ultraviolet-Visible Spectrophotometry Analysis

**Figure 2:**
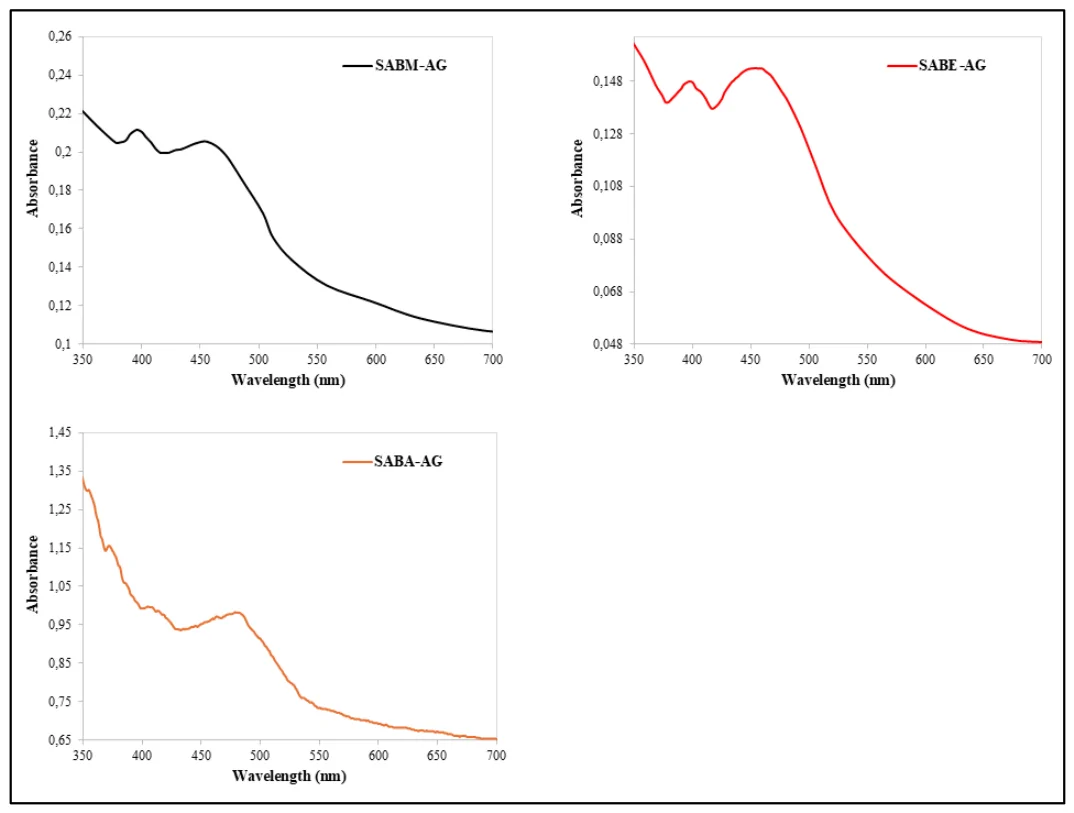
Ultraviolet-visible spectra of SA-AgNPs (2.68 mg/mL, 5 mM AgNO_3_) stored at 4 °C for 3 months; SABM-AG (bark methanol-AgNPs), SABE-AG (bark ethanol-AgNPs), SABA-AG (bark acetone-AgNPs), and SALE-AG (leaf ethanol-AgNPs).

UV-Vis spectrophotometry combined with SPR analysis confirmed the successful synthesis of SA-AgNPs. The SPR of electrons caused an increased peak intensity in the UV spectrum. SA-AgNPs produced from bark and leaf extracts (methanol, ethanol, and acetone) mixed with 5 mM AgNO_3_ exhibited distinct SPR peaks. Notably, both SABM-AG and SABE-AG showed double peaks at 397 nm and 454 nm, while SABA-AG displayed a narrow peak at 480 nm (Figure 2).

**Figure 3:**
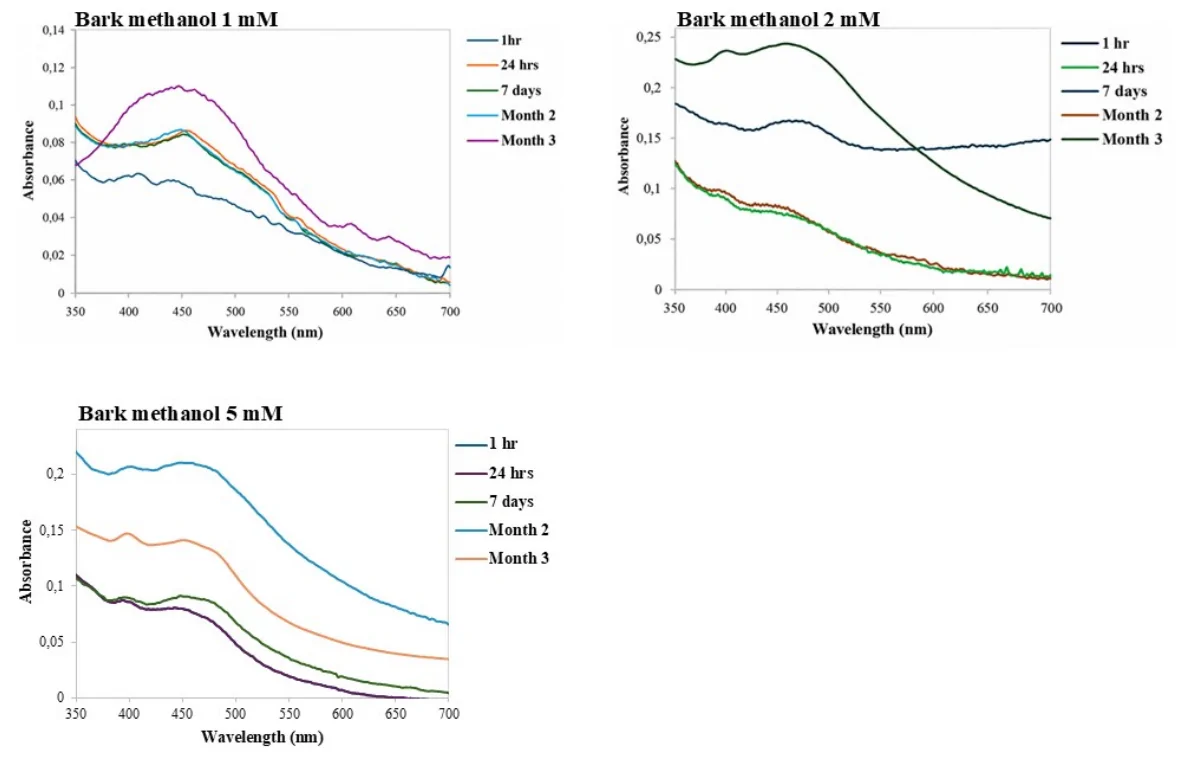
UV-Vis spectra of methanol extract-based SA-AgNPs, showing the effect of reaction time on nanoparticle synthesis. SA-AgNPs (2.68 mg/mL) were synthesized using AgNO_3_ concentrations of 1, 2, and 5 mM.

**Figure 4:**
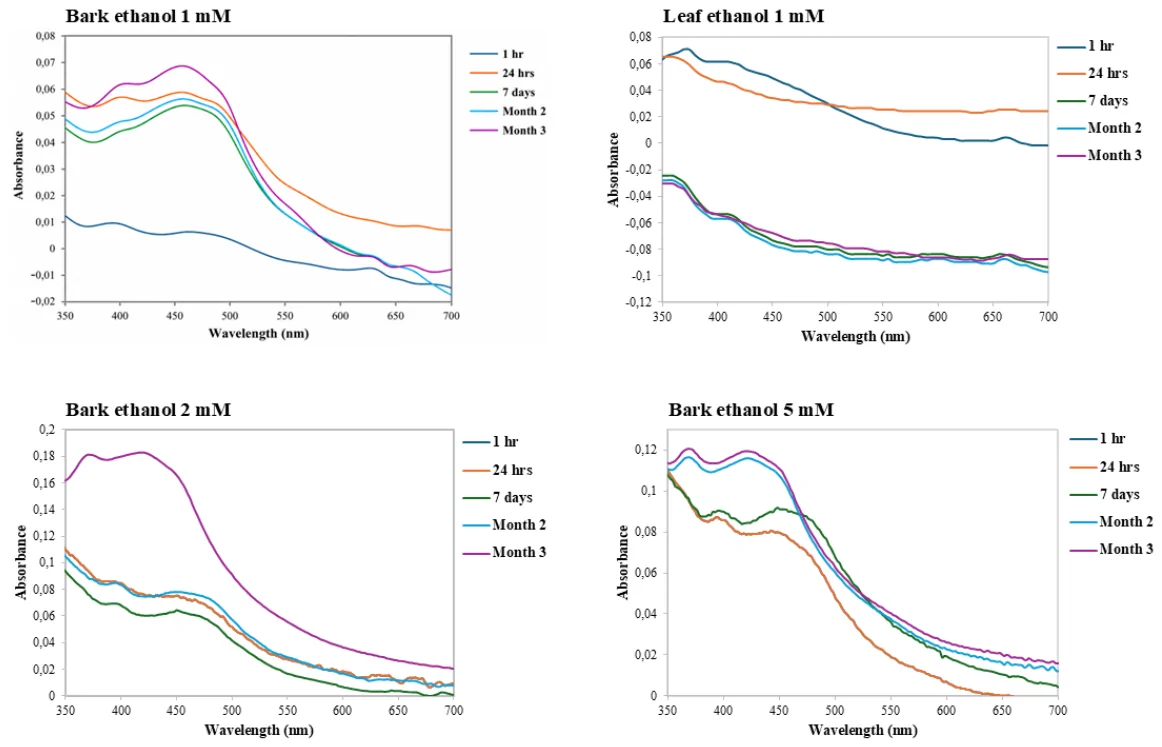
UV-Vis spectra of ethanol extract-based AgNPs showing the impact of reaction time. SA-AgNPs (2.68 mg/mL) were synthesized using AgNO_3_ concentrations of 1, 2, and 5 mM.

**Figure 5:**
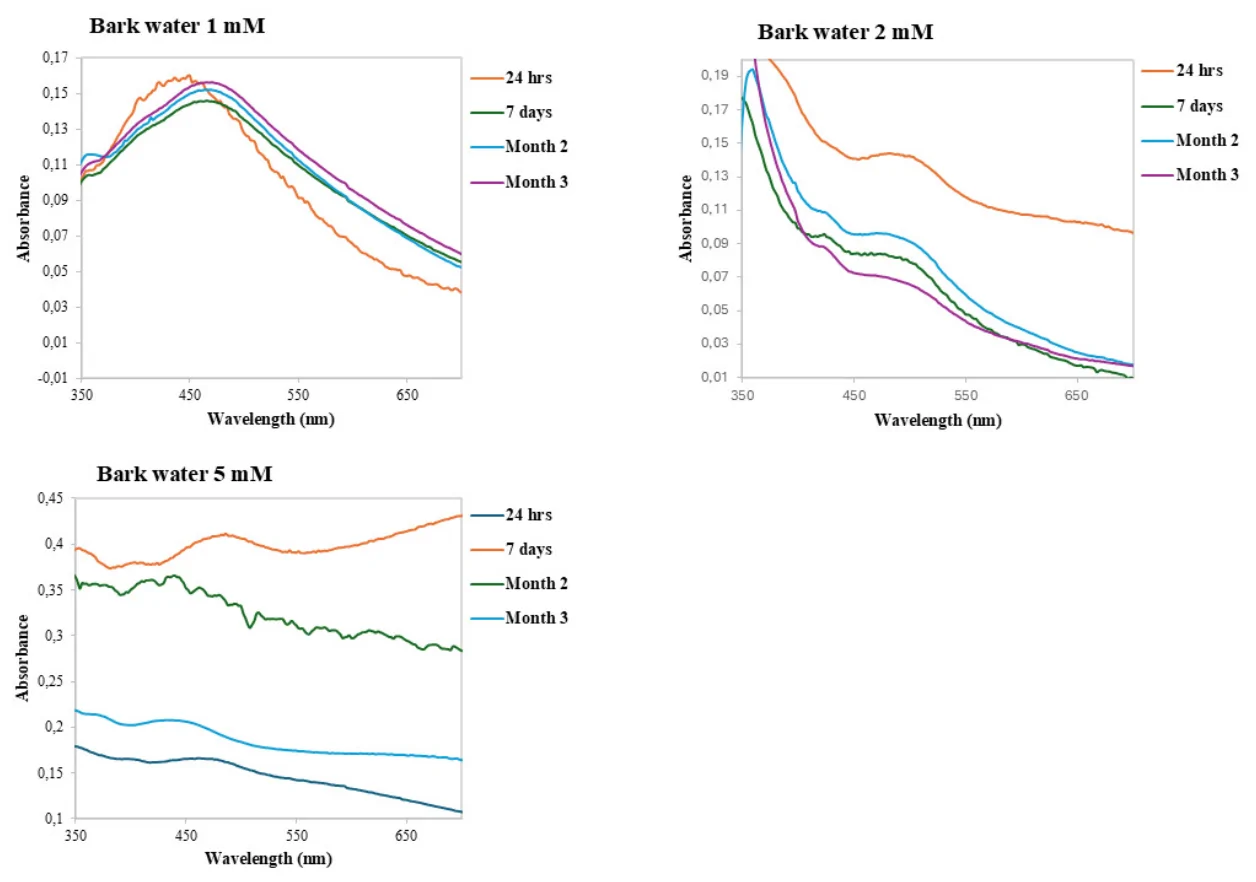
UV-Vis spectra of distilled-water extract-based AgNPs showing the impact of reaction time. SA-AgNPs (2.68 mg/mL) were synthesized using AgNO_3_ concentrations of 1, 2, and 5 mM.

The influence of reaction time on SA-AgNPs synthesis by UV-Vis spectral analysis is illustrated in Figure 3, Figure 4 and Figure 5. Sample SABM-AG/SALM-AG exhibited a decrease in their absorption peaks as the incubation time increased. For a 1 mM AgNO_3_ concentration, SPR peak was observed after 24 h at 454 nm on SABM-AG. The peaks became defined as the incubation period increased to month 3, with a peak at a wavelength of 450 nm. Meanwhile, 2 and 5 mM had more intense, narrower peaks, and the peaks formed within an hour. A well-defined SPR peak at 470 was observed after 7 days at both 2 and 5 mM concentrations on the SABM-AG extract. On the other hand, SALM-AG exhibited an absorption peak at 356 nm after 1 h, which remained constant up to 24 h. Thereafter, absorbance intensity gradually increased with increasing reaction time (Figure 3).

Figure 4 shows that SABE-AG/SALE-AG had similar trends to those observed with methanol conjugates, with SPR peaks decreasing as reaction time increased. SALE-AG exhibited strong peaks at 370 and 410 nm after 1 h, 7 days, and 2nd month at 1 mM concentration. While peaks at 364 nm were observed after 1 h for 2 and 5mM concentrations. Notably, 3rd month showed higher peaks compared to 2nd month and 7 days.

In contrast, SABD-AG formed after 24 h, with decreasing absorbance over time for 1 mM concentrations at both 356 and 468 nm (Figure 5). For the 2 mM AgNO_3_ concentration, SABD-AG had peaks at 360 after both 1 h and 24 h, which faded as the reaction time increased. At a 5 mM AgNO_3_ concentration, the SPR peak appeared after 1 h, intensifying by day 7 with peaks at 353, 404, and 485 nm. Leaf water (SALD-AG) samples exhibited an absorption peak at 380 nm after 24 h at 1 mM, with the absorbance fading over time (Figure S6). At 2 and 5 mM, no peaks were observed.

The impact of AgNO_3_ concentrations on the production of SA-AgNPs is shown in Figure 3, Figure 4 and Figure 5. The results revealed that as concentrations increased from 1 to 5 mM, color intensity increased and caused the SPR peak to shift towards higher wavelengths, suggesting NP aggregation. While increasing AgNO_3_ concentration from 1 to 2 mM resulted in higher absorbance values for SA-AgNPs, a decrease in absorbance was observed at 5 mM, indicating a transition point. However, this trend was not consistent across all samples; SABM-AG and SABA-AG exhibited increased absorption at 5 mM.

**Figure 6:**
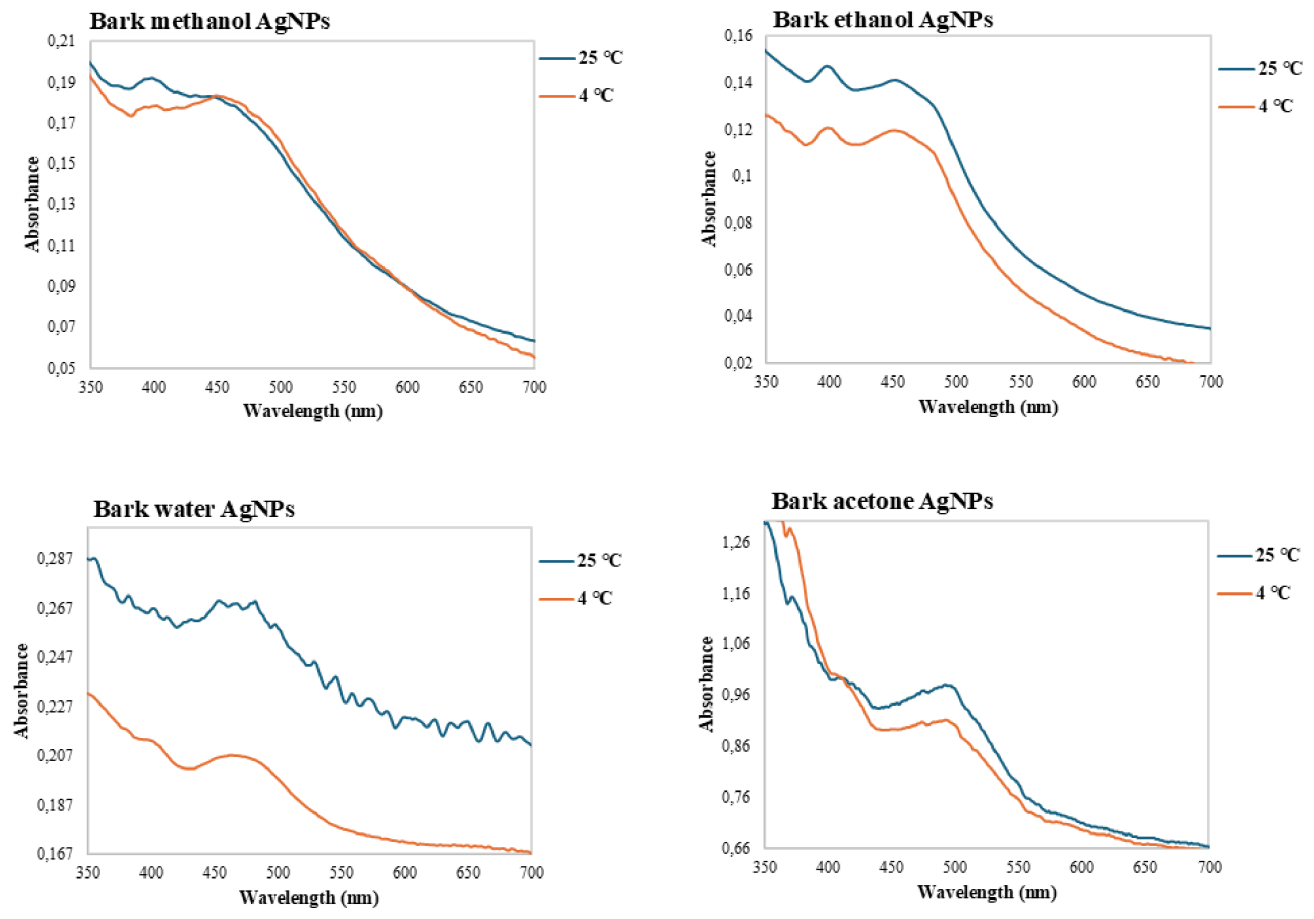
The influence of temperature on AgNPs synthesized by *S. africana* extracts. SA-AgNPs (2.68 mg/mL) were synthesized using AgNO_3_ concentrations of 5 mM.

Lastly, the influence of temperature on the synthesis of SA-AgNPs was studied, and the findings were presented in Figure 6. Results showed that increasing temperature led to heightened SPR peaks. Narrower bands with higher absorbance values were consistently observed at 25 °C compared to 4 °C across most samples, except for the SABD-AG (1 mM), which exhibited a slightly higher peak at 4 °C, and SALD-AG had no peaks (Figures S7–S9).

#### 3.2.2. X-Ray Diffraction Unit (XRD) Analysis of SA-AgNPs

**Figure 7:**
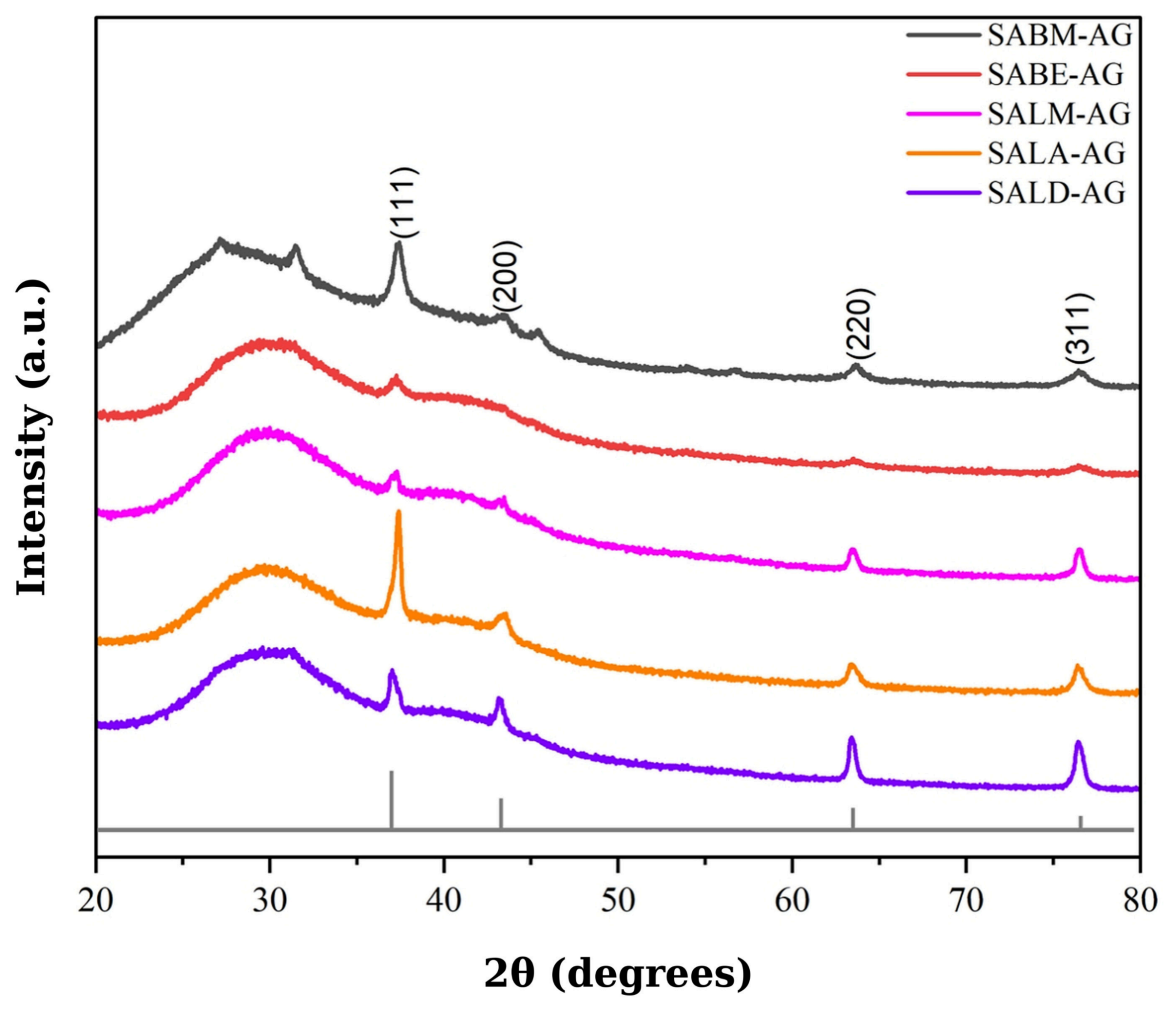
XRD spectrum of SA-AgNPs (2.68 mg/mL, 5 mM AgNO_3_, 4 °C) synthesized using *S. africana* extracts bark methanol-AgNPs (SABM-AG), bark ethanol-AgNPs (SABE-AG), leaf methanol-AgNPs (SALM-AG), leaf acetone-AgNPs (SALA-AG), and leaf water-AgNPs (SALD-AG).

X-ray diffraction (XRD) spectroscopy was used to characterize the crystallinity and structures of the SA-AgNPs. Four diffraction peaks were observed at 2θ values of 37°, 43°, 63°, and 76°, with planes at (111), (200), (220), and (311), respectively (Figure 7), using Bragg’s law. An additional peak at 45° was also observed for SAMB-AG, while SABE-AG did not display a peak at 2θ angle 43°. The average crystalline size of SA-AgNPs samples varied across different experimental conditions, ranging from 9 to 19 nm, and is summarized in Table 1.

#### 3.2.3. Transmission Electron Microscopy (TEM) Analysis of SA-AgNPs

**Figure 8:**
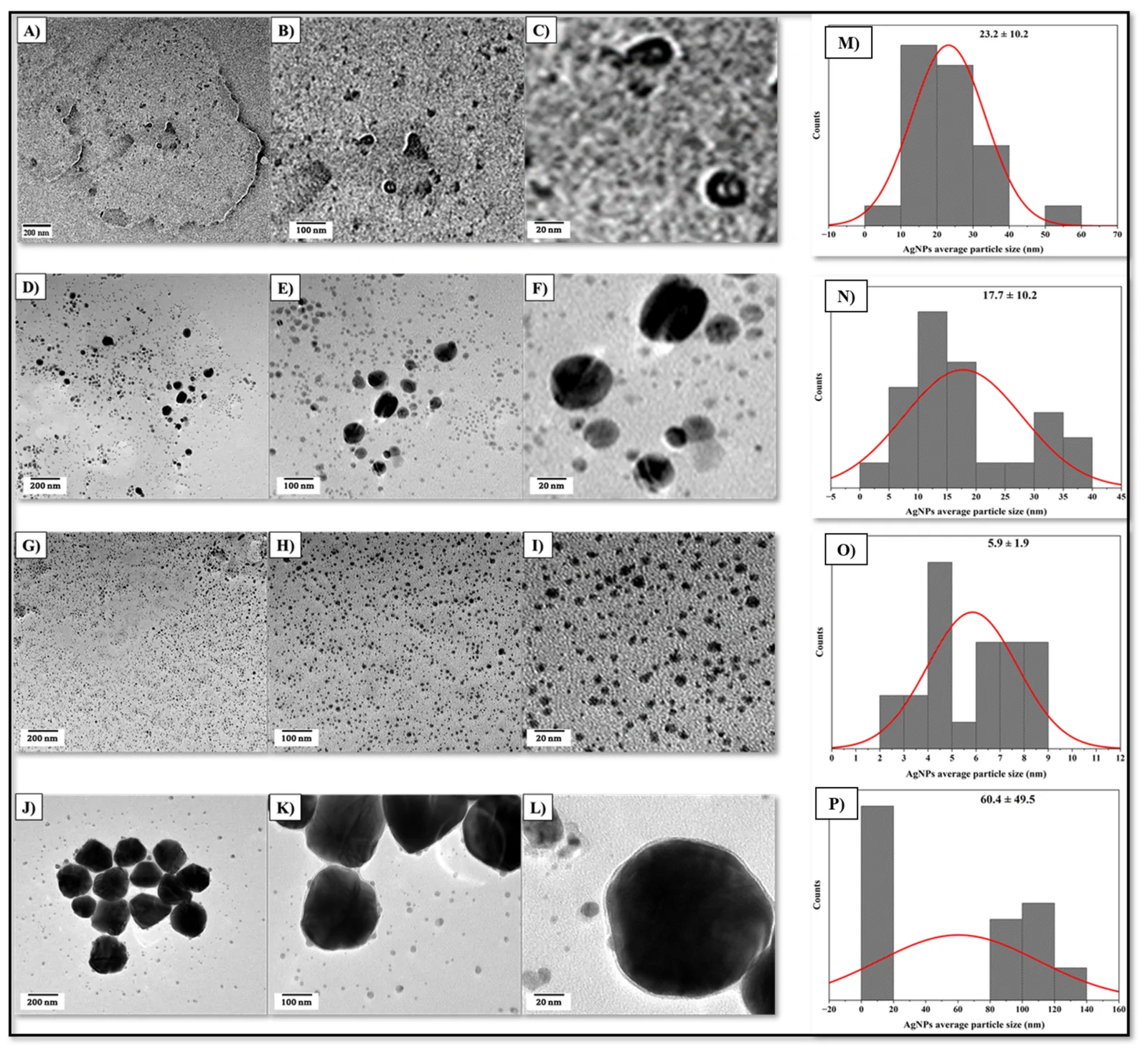
The TEM micrographs of various samples of SA-AgNPs (2.68 mg/mL, 5 mM AgNO_3_, 4 °C) at different magnifications (200, 100, and 20 nm) and their corresponding size distributions displayed in histograms. From (**A**–**C**) = SABM-AG (bark methanol-conjugated AgNPs), (**D**–**F**) = SABE-AG (bark ethanol-conjugated AgNPs), (**G**–**I**) = SABA-AG (bark acetone-conjugated AgNPs), and (**J**–**L**) = SALE-AG (leaf ethanol-conjugated AgNPs). The corresponding particle-size distribution histograms are (**M**) SABM-AG, with an average particle size of 23.2 ± 10.2 nm; (**N**) SABE-AG, 17.7 ± 10.2 nm; (**O**) SABA-AG, 5.9 ± 1.9 nm; and (**P**) SALE-AG, 60.4 ± 49.5 nm.

Transmission electron microscopy (TEM) was used to investigate the shape and size of SA-AgNPs. Figure 8 displays TEM micrograph images of SA-AgNPs at different magnifications (200, 100, and 20 nm), along with their corresponding particle size distribution portrayed in histogram graphs. The TEM analysis revealed diverse morphologies and sizes of both types of NPs. It was observed that SA-AgNPs consisted of smaller particles predominantly exhibiting spherical shapes, along with larger particles displaying squashed-spherical, hexagonal, and irregular forms.

The average core size of SA-AgNPs exhibited significant variation across samples. Specifically, the SABM-AG (A–C), SABE-AG (D–F), SABA-AG (G–I), and SALE-AG (J–L) samples showed particle size ranges of 13 to 53 nm, 4 to 35 nm, 2 to 9 nm, and 5 to 136 nm, respectively. The corresponding average particle size distribution was 23.2 ± 10.2, 17.7 ± 10.2, 5.9 ± 1.9, and 60.4 ± 49.5 nm, indicating a notable diversity in size among the various samples.

#### 3.2.4. Fourier-Transform Infrared Spectroscopy (FTIR) Analysis of the Plant Extracts and Conjugated Nanoparticles

**Figure 9:**
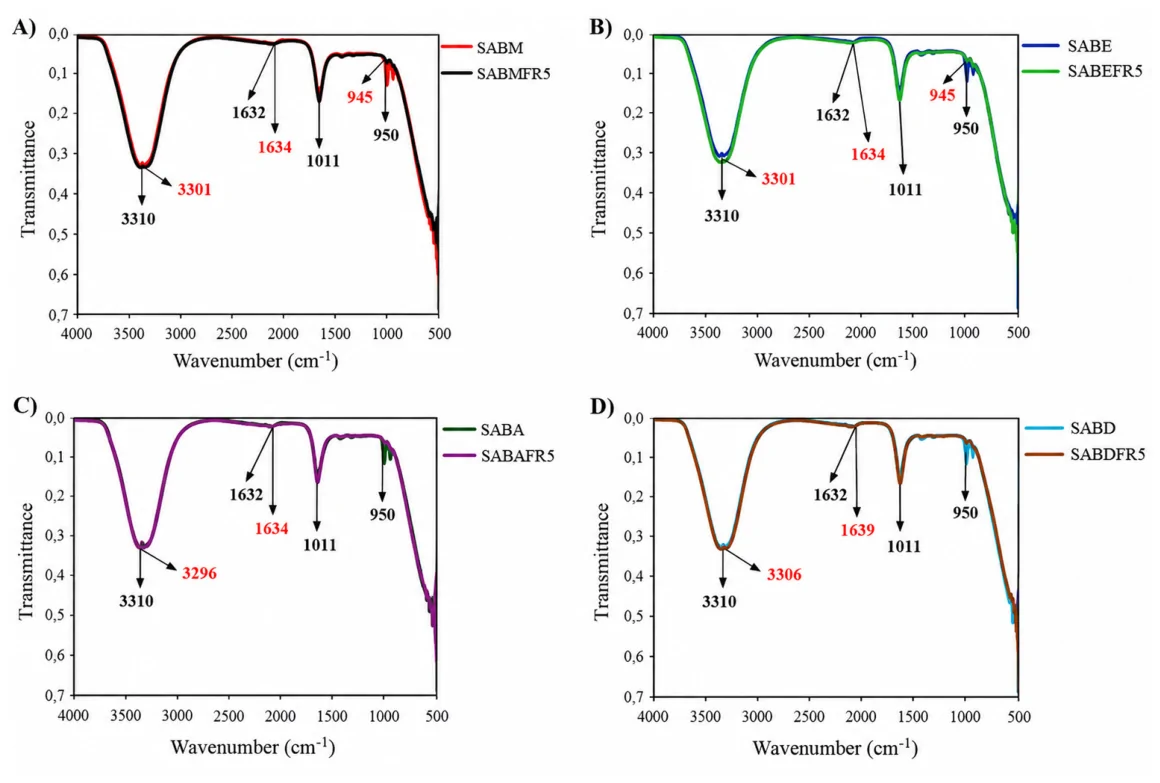
FTIR spectra of *S. africana* extracts (10 mg/mL) and their synthesized SA-AgNPs (2.68 mg/mL, 5 mM AgNO_3_, 4 °C): (**A**) bark methanol extract (SABM) and methanol-AgNPs (SABM-AG), (**B**) bark ethanol extract (SABE) and ethanol-AgNPs (SABE-AG), (**C**) bark acetone extract (SABA) and acetone-AgNPs (SABA-AG), (**D**) bark water extract (SABD) and water-AgNPs (SABD-AG). Red numbers indicate the shifted FTIR absorption bands of the synthesized AgNPs relative to those of the corresponding crude extracts.

FTIR spectral analysis of *S. africana* crude extracts confirmed key functional groups (Figure 9A–D). Bark extracts showed a broad peak at 3310 cm^−1^, indicating O-H (hydroxy) groups, and a narrow peak at 1632 cm^−1^, corresponding to C=C (alkenes) or N-H (amide) groups. Additional absorption peaks were observed at 1011 cm^−1^ (C-O stretching, alcohols) and 950 cm^−1^ (C-H aromatic in-plane bending) [35,36].

FTIR spectra of SA-AgNPs displayed similar functional groups with minor shifts. The broad peak shifted differently among samples: SABA-AG (3296 cm^−1^), SABM-AG and SABE-AG (3301 cm^−1^), and SABD-AG (3306 cm^−1^). The narrow peak shifted slightly to 1634 cm^−1^ for all SA-AgNPs. The peak at 1011 cm^−1^ remained unchanged. The last peak shifted to 945 cm^−1^ in SABM-AG and SABE-AG, while remaining at 950 cm^−1^ in SABM-AG and SABD-AG.

### 3.3. Antimicrobial Activity

The antimicrobial activities of *S. africana* extracts and their synthesized SA-AgNPs were tested against the WHO priority bacterial pathogens (*A. baumannii*, *P. aeruginosa*, *E. coli*, and *S. aureus*), responsible for RTIs. The effectiveness and stability of SA-AgNPs were assessed for three months, and the results presented in Experiment 1 corresponded to the activity exhibited in the first month, Experiment 2 in the second month, and Experiment 3 in the third month. Our findings indicated that SA-AgNPs synthesized with a 5 mM AgNO_3_ concentration and incubated at 4 °C exhibited the best antimicrobial activity. In comparison, those synthesized with 1 mM and 2 mM concentrations, as well as those stored at 25 °C, showed inferior antimicrobial effectiveness.

#### 3.3.1. Agar Well-Diffusion Assay

**Figure 10:**
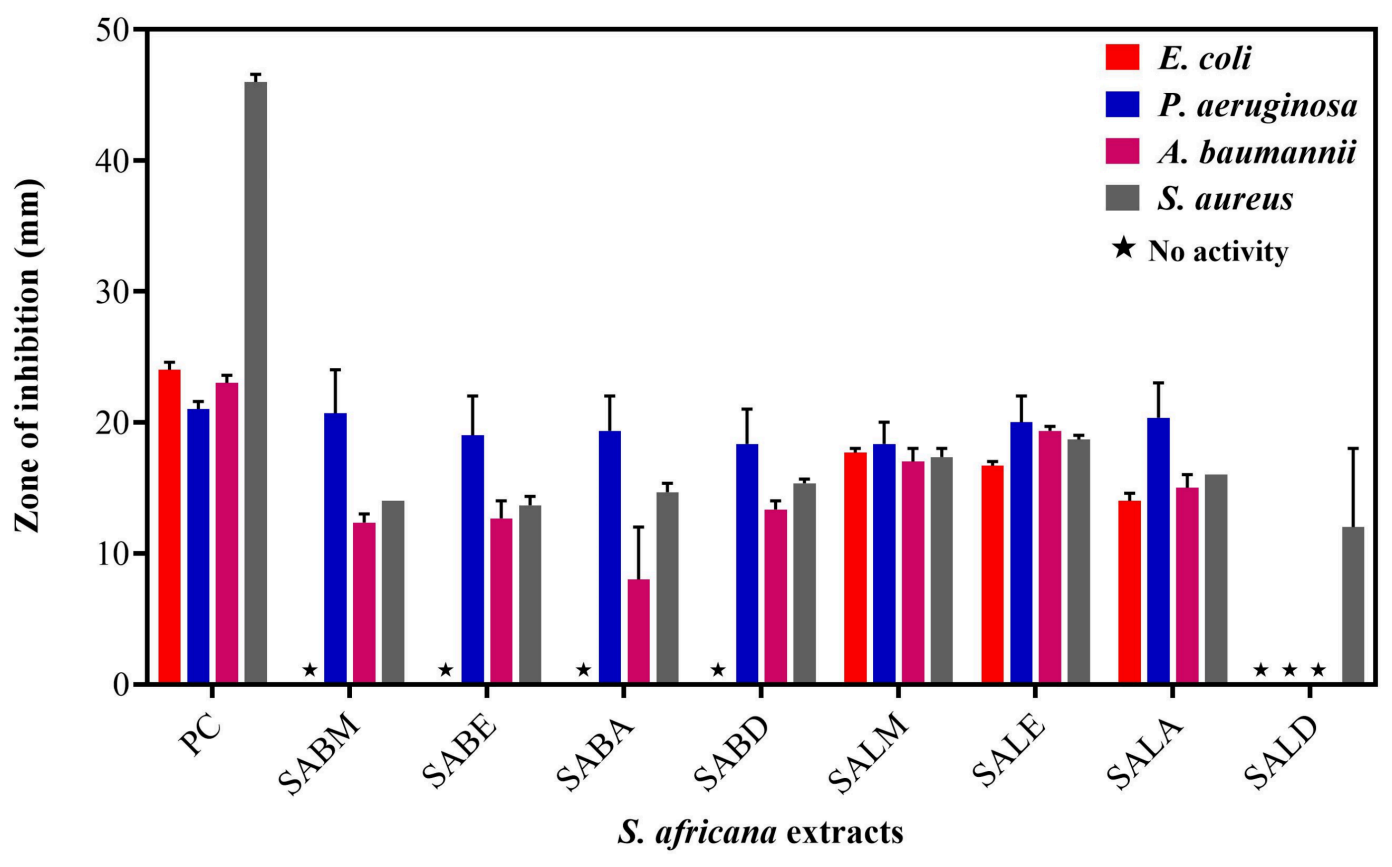
Zone of inhibition (mm) of *S. africana* plant extracts (10 mg/mL) against selected bacteria. SABM = bark methanol, SABE = bark ethanol, SABA = bark acetone, SABD = bark water, SALM = leaf methanol, SALE = leaf ethanol, SALA = leaf acetone, SALD = leaf water. PC = positive control (Lomefloxacin hydrochloride), NC = negative control (10% Dimethyl sulfoxide). Data represent mean ± SEM (n = 3) (One-way ANOVA, Tukey’s post hoc test, *p* < 0.05).

The antimicrobial activities of *S. africana* crude extracts showed a significant difference compared to the positive control (one-way ANOVA, *p* = 0.0395), with Tukey’s post hoc test indicating significant differences between specific treatment means (*p* < 0.05). Agar well-diffusion assays revealed that the extracts were most active against *P. aeruginosa*, followed by *A. baumannii* and *S. aureus*. Bark extracts and water leaf extracts showed no activity against *E. coli* (Figure 10). Specifically, the highest inhibition was observed with the SABM (21 mm) against *P. aeruginosa*, followed closely by SALE and SALA, each exhibiting 20 mm inhibition zones against *P. aeruginosa*. SALE also showed 19 mm zones of inhibition against both *A. baumannii* and *S. aureus*. Water extracts generally exhibited lower activity across all bacteria, with SALD showing no inhibition except minimal activity against *S. aureus*.

**Figure 11:**
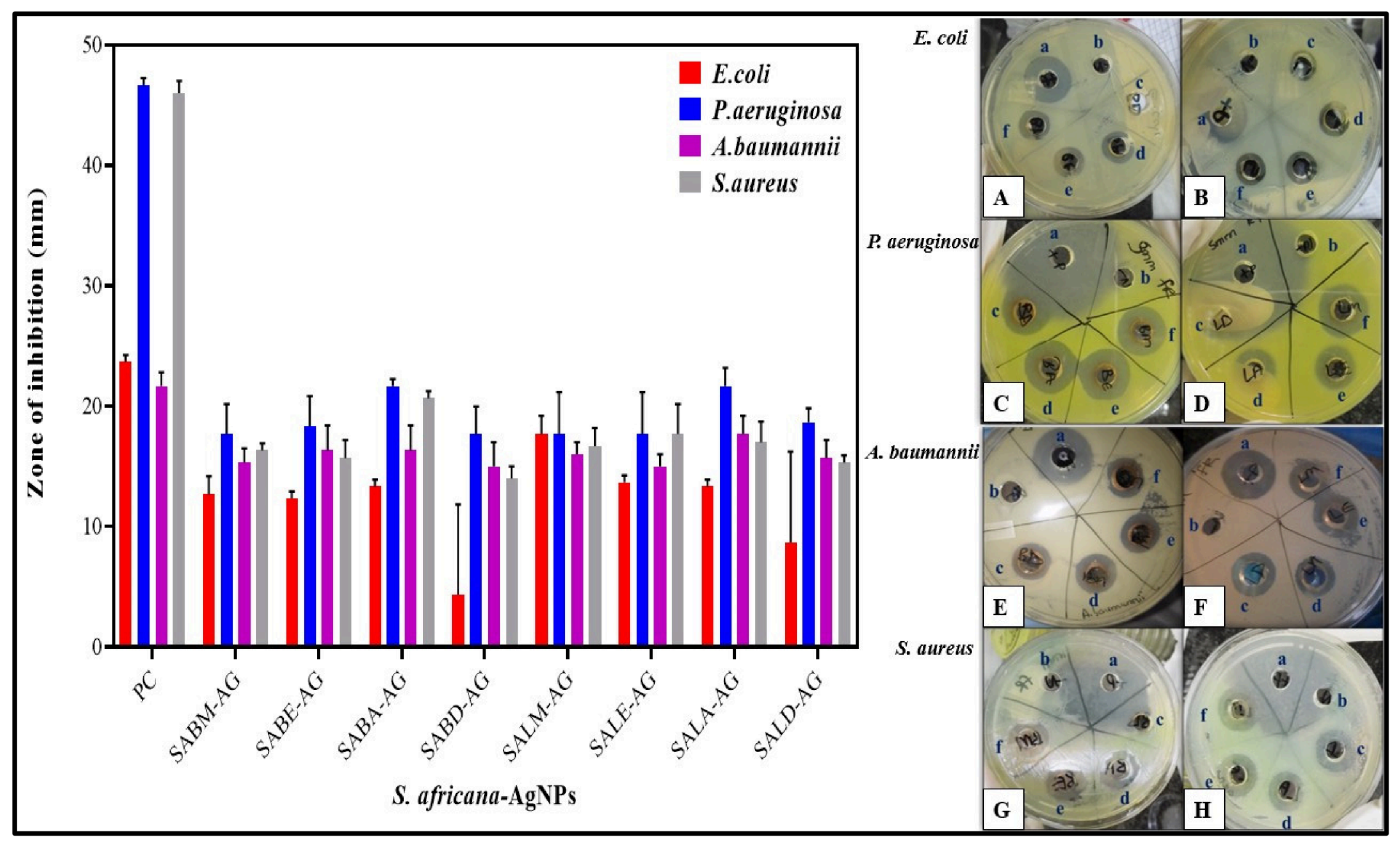
Antimicrobial activity of SA-AgNPs (2.68 mg/mL, 5 mM AgNO_3_, 4 °C) by zone of inhibition (mm) against *E. coli* (**A**,**B**), *P. aeruginosa* (**C**,**D**), *A. baumannii* (**E**,**F**), and *S. aureus* (**G**,**H**). Bark methanol (SABM-AG), bark ethanol (SABE-AG), bark acetone (SABA-AG), and bark water (SABD-AG) extracts. Leaf methanol (SALM-AG), leaf ethanol (SABE-AG), leaf acetone (SABA-AG), and leaf water (SALD-AG); a = positive control (PC; Lomefloxacin hydrochloride), b = negative control (10% Dimethyl sulfoxide). Data represent mean ± SEM (n = 3) (One-way ANOVA, Tukey’s post hoc test, *p* < 0.05).

Figure 11 presents the zone of inhibition in both a bar graph on the left and agar plates on the right. Among the tested strains, the SA-AgNPs exhibited greater efficacy against *P. aeruginosa*, followed by *S. aureus* and *A. baumannii*, and demonstrated relatively less activity against *E. coli*. Notably, SABA-AG and SALA-AG displayed the highest inhibition against *P. aeruginosa* (22 mm). SABA-AG showed the greatest inhibition against *S. aureus* (21 mm). Against *A. baumannii*, SALA-AG was most effective with an 18 mm zone, while *E. coli* was more susceptible to SALM-AG, which showed 18 mm inhibition. These differences were statistically significant (*p* = 0.0346) compared to the positive control.

Conversely, SABD-AG showed lower activity with no inhibition of *E. coli* and weak effects against *P. aeruginosa* and *S. aureus*. SALE-AG was also less active, particularly against *A. baumannii*. Overall, the results highlight variable antimicrobial activities among the samples, with SABA-AG and SALA-AG being the most potent against the tested pathogens.

#### 3.3.2. Microdilution Assay

**Figure 12:**
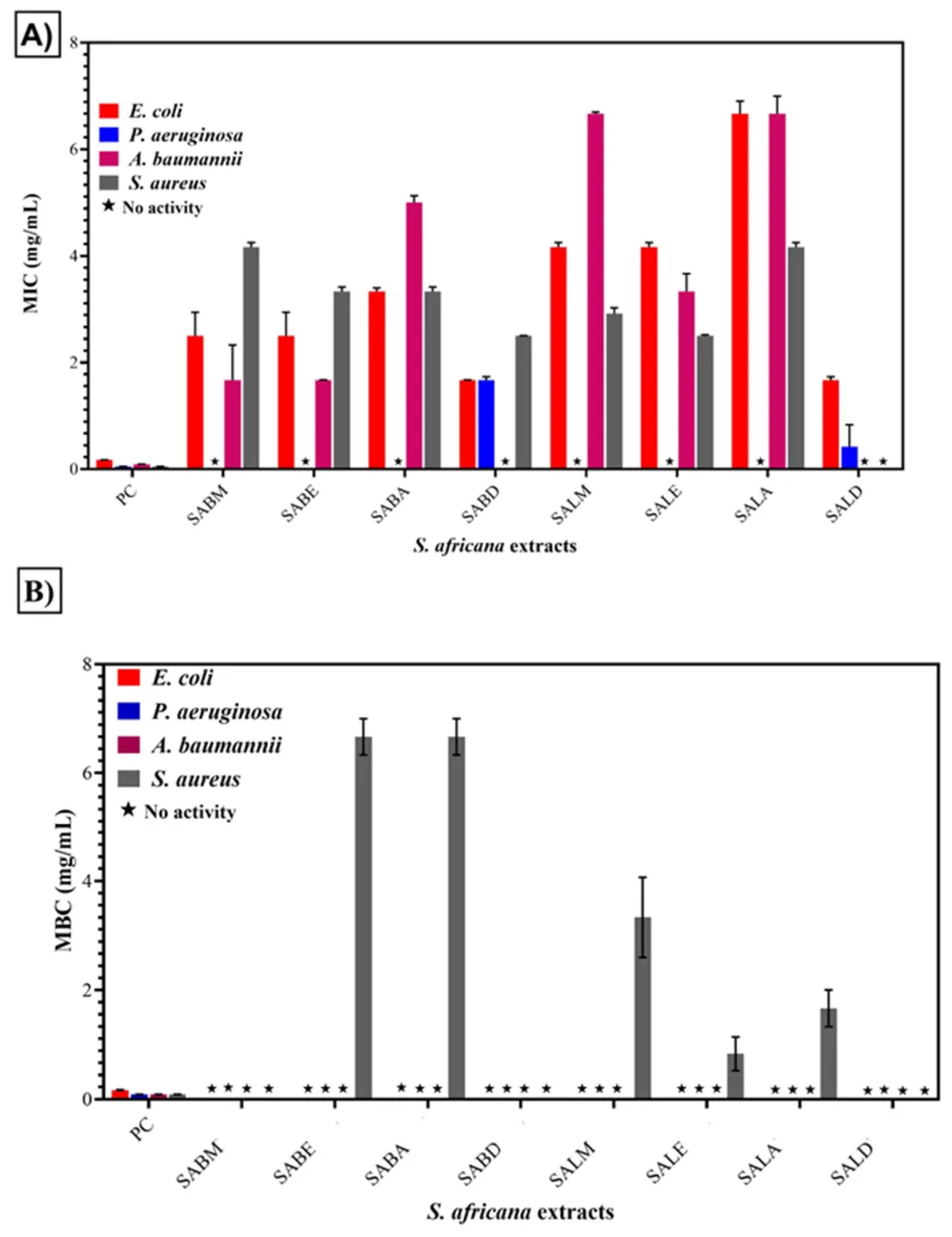
MIC (**A**) and MBC (**B**) of *S. africana* plant extracts (10 mg/mL) against selected bacteria. SABM = bark methanol, SABE = bark ethanol, SABA = bark acetone, SABD = bark water, SALM = leaf methanol, SALE = leaf ethanol, SALA = leaf acetone, SALD = leaf water. PC = positive control (Lomefloxacin hydrochloride), NC = negative control (10% Dimethyl sulfoxide). Data are presented as SEM (n = 3) (One-way ANOVA, Tukey’s post hoc test, *p* > 0.05).

The MIC results of *S. africana* extracts showed *E. coli* susceptibility to all extracts, followed by *S. aureus* and *A. baumannii*, except for SALD. *P. aeruginosa* was susceptible only to SABD and SALD, with the lowest MIC observed in SALD (0.42 mg/mL). SABD exhibited MICs of 1.67 mg/mL against both *P. aeruginosa* and *E. coli*. SABM and SABE showed MICs of 1.67 mg/mL against *A. baumannii*. The least active extracts were SALM and SALA against *A. baumannii*, and SALA against *E. coli*, each with an MIC of 6.67 mg/mL (Figure 12A).

The MBC results showed that the plant extracts exhibited bactericidal activity only against *S. aureus* (Figure 12B). The extract with the strongest killing activity was SALE, with the lowest MBC of 0.83 mg/mL. SABM, SABD, and SALD showed no killing activity against *S. aureus*. No extracts showed bactericidal effects against *E. coli*, *P. aeruginosa*, or *A. baumannii*. Differences in both MIC and MBC values compared to the control were not statistically significant (*p* = 0.1322 and *p* = 0.3971, respectively), suggesting that these variations may be due to chance rather than true antimicrobial effects.

**Figure 13:**
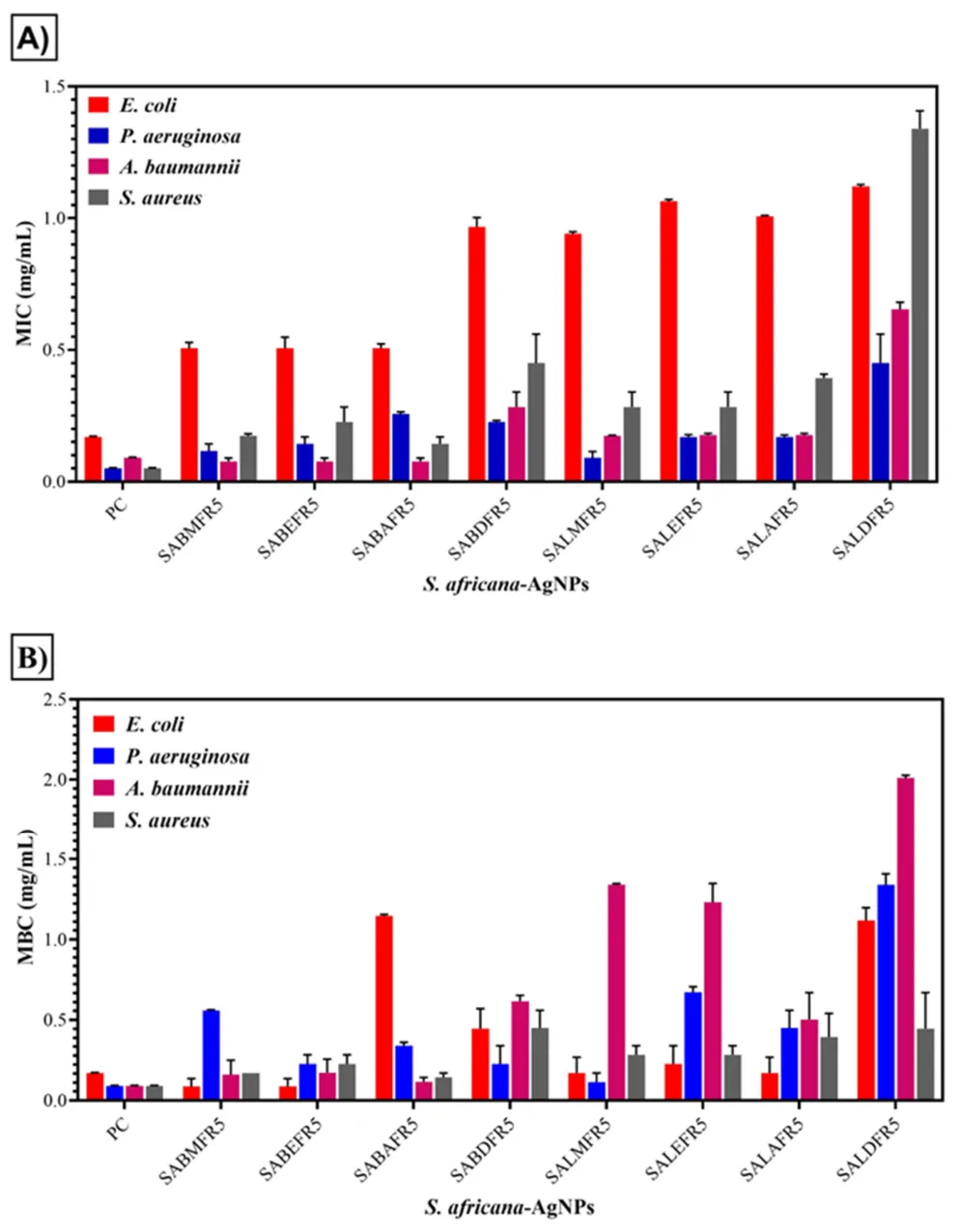
MIC (**A**) and MBC (**B**) of SA-AgNPs (2.68 mg/mL, 5 mM AgNO_3_, 4 °C) synthesized from bark methanol (SABM-AG), bark ethanol (SABE-AG), bark acetone (SABA-AG), bark water (SABD-AG), leaf methanol (SALM-AG), leaf ethanol (SALE-AG), leaf acetone (SALA-AG), and leaf water (SALD-AG) extracts. PC = positive control (Lomefloxacin hydrochloride); NC = negative control (10% dimethyl sulfoxide). Data represent mean ± SEM (n = 3). One-way ANOVA, Tukey’s post hoc test, *p* < 0.05 for MIC and *p* > 0.05 for MBC.

The MIC results for SA-AgNPs highlighted varying levels of activity among the samples against the tested bacterial strains (Figure 13A). The antimicrobial activity of SA-AgNPs was statistically significant compared to the control (*p* = 0.0273), indicating the reliability of their effects. SABM-AG, SABE-AG, and SABA-AG exhibited the lowest MIC value of 0.08 mg/mL against *A. baumannii*. SALM-AG demonstrated a MIC of 0.09 mg/mL against *P. aeruginosa*, while SABA-AG showed a MIC of 0.14 mg/mL against *S. aureus*. Conversely, SABD-AG and SALD-AG exhibited the least potent antimicrobial effects, with SALD-AG displaying a notably high MIC of 1.34 mg/mL against *S. aureus*. *E. coli* was the least susceptible.

The MBC results showed varying bactericidal activity among the SA-AgNP samples against the tested strains. SABM-AG and SABE-AG exhibited the lowest MBC of 0.1 mg/mL against *E. coli*, indicating strong bactericidal effects. SALM-AG showed activity against *P. aeruginosa* with an MBC of 0.11 mg/mL. SABA-AG demonstrated MBCs of 0.12 mg/mL and 0.14 mg/mL against *A. baumannii* and *S. aureus*, respectively. In contrast, SALD-AG displayed the least activity, with MBC values ranging from 0.45 to 2 mg/mL. The bactericidal activity showed no significant difference (*p* = 0.0960), suggesting that the killing effects might be due to random variation (Figure 13B).

## 4. Discussion

Medicinal plants have a long history of use against various diseases, including RTIs [37]. Their antimicrobial potential is largely due to diverse secondary metabolites. The global rise of ABR bacteria has led to a global crisis, prompting increased interest in medicinal plants as sources of novel antibacterial agents [38]. Plant-based AgNPs have gained recognition as eco-friendly and effective alternatives for combating ABR bacteria [39].

In this study, AgNPs were synthesized using methanol, ethanol, acetone, and water extracts of *S. africana*, and their antimicrobial efficacy was evaluated against WHO-priority ABR bacteria associated with RTIs. SA-AgNPs synthesized with 5 mM AgNO_3_ and incubated at 4 °C exhibited the strongest antimicrobial activity. This aligns with López-Miranda et al. [40] and Htwe et al. [41], who found that 5 mM AgNO_3_ favors NP formation with narrow size distributions and enhanced activity.

A progressive color change from light yellow/brown at 1 mM to darker shades at higher AgNO_3_ concentrations (2–5 mM) suggests NP agglomeration influenced by metal ion concentration, consistent with previous findings [42]. Maintaining AgNPs under low temperature and darkness improved their long-term stability by preventing aggregation while preserving morphology and size distribution, as demonstrated by Velgosova et al. [43]. This was corroborated in our stable SA-AgNPs stored at 4 °C for over three months.

The kinetics of NP formation is influenced by initial silver ion concentration; higher concentrations accelerate Ag^+^ reduction to metallic Ag^0^, consistent with our observation of rapid color change within an hour at 5 mM AgNO_3_ [41,44]. The UV-Vis spectroscopy confirmed SA-AgNP synthesis by characteristic surface plasmon resonance (SPR) absorbance peaks, reflecting collective oscillations of conduction electrons responsive to visible light, which sensitively depend on NP size, shape, and distribution [45].

Samples (SABM-AG/SABE-AG, and SALA-A) exhibited dual SPR peaks indicating anisotropic (non-spherical) morphologies and polydispersity [46,47,48]. The peak at shorter wavelengths (370–397 nm) typically corresponds to smaller, spherical or near-spherical particles exhibiting transverse SPR modes, whereas the longer wavelength peak (454–500 nm) arises from larger particles, aggregated species, or different plasmon modes such as longitudinal or in-plane resonances characteristic of anisotropic shapes like rods or plates. This dual-peak phenomenon is consistent with the literature reporting SPR bands within 350–550 nm for AgNPs of diversified shapes and sizes [49].

In contrast, samples with single (SALM-AG and SALE-AG) sharp peaks indicated small monodisperse, spherical NPs. Similarly, plant-based AgNPs with peaks around 390 nm have been reported to contain evenly distributed small spherical particles [50]. Additionally, a narrow SPR peak at 480 nm for sample (SABA-AG), correlating with relatively homogeneous NPs in size and distribution, a finding supported by Ahmad et al. [51] and other studies reporting similar absorbance around this wavelength [52,53]. The optical color closely correlated with NP size and dispersion. The smaller, well-dispersed particles generated transparent yellow or light brown solutions, while larger or aggregated particles caused red-shifted, turbid solutions. Decreased transparency and visible precipitation in Falcon tubes over incubation time reflected aggregation and reduced colloidal stability.

The influence of reaction time on the synthesis of SA-AgNPs showed that methanol and ethanol extracts exhibited decreased SPR peak intensity with prolonged incubation, accompanied by the SPR peak shifting towards a higher wavelength region (red shift). These spectral changes indicate large NP size and aggregation over time. These align with previous studies demonstrating that the appearance of characteristic SPR bands corresponds to the formation of AgNPs [49,54,55]. Temperature effects were also significant; at 25 °C, SPR peak intensity and sharpness were greater than at 4 °C, reflecting enhanced reduction kinetics and the formation of larger or more aggregated NPs at higher temperatures. This is consistent with Stavinskaya et al. [56], who demonstrated that elevated temperatures accelerate the reduction of Ag^+^ ions, facilitating faster NP synthesis.

The characterization of SA-AgNPs by XRD confirmed their crystalline nature, with distinct peaks corresponding to the face-centered cubic silver structure (JCPDS No. 01-1167) [57,58]. The observed peak broadening and variation in intensities suggest anisotropic growth and nanocrystal sizes ranging from 9 to 19 nm, consistent with biosynthetic variability. An additional peak at 45° in some samples (SABM-AG) likely indicates residual organic compounds from *S. africana* extracts acting as capping agents [58]. This observation is supported by findings from Salayová et al. [59], who also noted variations in peak characteristics that could be attributed to organic compounds from plant extracts. Another interesting observation in all samples is that the lattice planes corresponding to (200), (220), and (311) were noted to exhibit weak intensity and broadened peaks compared to the (111) Bragg’s reflection. This suggests that the nanocrystals synthesized biologically display a high level of anisotropy, with the NPs predominantly oriented along the (111) direction [60].

TEM analysis revealed a polydisperse population with varying morphologies, including small spherical particles and larger squashed-spherical, hexagonal, and irregular shapes, with core sizes ranging from approximately 6 to 60 nm across samples, which is typical for plant-mediated syntheses influenced by phytochemical composition. Differences in phytochemical composition between the extracts may influence the nucleation, growth, surface capping, and stabilization of the nanoparticles, resulting in variations in particle size and morphology [61]. Notably, smaller particles, such as SABA-AG with an average size around 6 nm, correlated with enhanced biological activity.

The difference between the XRD crystallite sizes and TEM particle sizes can be attributed to the different structural parameters measured by the two techniques. XRD estimates the size of coherently diffracting crystalline domains, whereas TEM provides direct information on the physical dimensions and morphology of the particles. Therefore, crystallite size and particle size are not necessarily equivalent [62]. This is demonstrated by Bhakya et al., who reported TEM particle sizes of 16–95 nm compared with an XRD crystallite size of 38.23 nm [32]. Conversely, close agreement between the two measurements has also been reported, demonstrating that the relationship depends on the crystallinity, morphology, and aggregation state of the nanoparticles [63]. Thus, the XRD and TEM results obtained in the present study should be regarded as complementary rather than directly equivalent measurements.

FTIR detected functional groups such as hydroxyl, alkene/amide, alcohol, and aromatic C-H groups in *S. africana* extracts, with shifts in absorption bands upon SA-AgNPs formation suggesting interaction with silver ions. The variation in peak shifts among SA-AgNP samples reflects differences in extract composition or binding strength, which may influence NP properties and antimicrobial activity. These results imply that the phytochemicals present in the *S. africana* extracts, such as polyphenols, flavonoids, and terpenoids, are likely involved in the reduction and capping of the NPs [64,65]. Corresponding studies with other plant extracts, such as *D. kaki* and *E. senticosus*, have also shown the involvement of functional groups like hydroxyl, carboxyl, and amine groups in the reduction and capping of Ag+ NPs [66,67]. The shifting of the C=O peak in the FTIR spectra obtained from AgNPs indicates that carbonyl-containing functional groups may be responsible for the bioreduction of Ag^+^ ions into AgNPs, while the shifting of stretching vibrations of O-H and N-H to lower wavelengths suggests binding of these functional groups with the surface of the AgNPs [67].

Antimicrobial assays demonstrated pronounced inhibitory effects of SA-AgNPs, particularly acetone-extract conjugates, against *P. aeruginosa* and *S. aureus*, consistent with previous studies where AgNPs inhibit both Gram-negative and Gram-positive bacteria, including multidrug-resistant strains [68,69]. Crude plant extracts showed markedly lower activities, highlighting the enhanced bioavailability, cellular uptake, and bactericidal efficiency afforded by nanoformulations, as documented in other plant-based synthesized AgNP studies [70,71]. Zones of inhibition reached up to 22 mm against *P. aeruginosa* (SABA-AG) and 21 mm against *S. aureus* (SABA-AG), highlighting potent antibacterial efficacy. This supports the critical role of AgNO_3_ concentration and phytochemical capping in activity. These findings align with earlier research on the antibacterial effects of plant-derived AgNPs by agar well-diffusion methods and revealed significant activity against *P. aeruginosa*, suggesting that the efficacy of these NPs depends on their 5 mM AgNO_3_ concentration [59]. Conversely, Liaqat et al. [25] reported higher activity of plant-based AgNPs against Gram-positive bacteria, suggesting differential susceptibility due to outer membrane lipopolysaccharides impeding NP penetration in Gram-negatives [72].

Proposed antibacterial mechanisms include membrane disruption through interaction with phospholipids and proteins, causing permeability increases and cellular content leakage [73]. The generation of reactive oxygen species (ROS) induces oxidative stress, damaging vital biomolecules, explaining the heightened sensitivity of *P. aeruginosa* in our results. Released silver ions further enhance inhibition by disrupting metabolic processes [74,75].

Microdilution assays confirmed significant antibacterial activity of SA-AgNPs compared to extracts alone. Bark methanol, ethanol, and acetone (SABM-AG, SABE-AG, SABA-AG) demonstrated potent activity against *Acinetobacter baumannii* (MIC as low as 0.08 mg/mL), while methanol (SALM-AG) was active against *P. aeruginosa* (0.09 mg/mL). Gram-negative bacteria generally showed higher susceptibility, likely due to structural differences in cell envelopes facilitating nanoparticle penetration. SABA-AG showed activity (MIC of 0.14 mg/mL) against *S. aureus*. However, water extracts conjugated with AgNPs displayed lower efficacy against all tested strains. The MBC results highlighted potent bactericidal effects for samples such as SABM-AG and SABE-AG against *E. coli* (MBC of 0.1 mg/mL) and SALM-AG against *P. aeruginosa* (MBC of 0.11 mg/mL). Mohammed [76] stated that the antibacterial efficacy of AgNPs is derived from their ability to disrupt microbial membranes containing adhesive substances like proteins and polysaccharides, as well as the bactericidal effects of Ag^+^ ions.

Reports indicate that extract-based AgNPs exhibit enhanced antibacterial activity compared to the extracts alone [77]. The combination of AgNPs with plant phytochemicals produces synergistic effects, making them promising agents against ABR bacteria. SA-AgNPs demonstrated superior antimicrobial efficacy owing to the sustained release of silver ions that disrupt bacterial cell walls, coupled with secondary metabolites adsorbed on the NP surface that enhance bacterial membrane interaction [59,78]. Smaller NPs, with larger surface areas, facilitate better contact and penetration, resulting in greater bactericidal effects. These findings demonstrate the antibacterial potential of *S. africana*-derived AgNPs against the tested bacterial strains. However, their therapeutic applicability cannot be established based on antibacterial activity alone, and further cytotoxicity, hemolysis, mechanistic, and in vivo safety studies are required.

## 5. Conclusions

This study successfully demonstrated the green synthesis of silver nanoparticles from *S. africana* extracts using methanol, ethanol, acetone, and water. Of all the samples, bark methanol (SABM-AG), ethanol (SABE-AG), and acetone extracts (SABA-AG, SALA-AG) conjugated AgNPs exhibited potent antibacterial activity, particularly against WHO priority pathogens including *P. aeruginosa* and *S. aureus*, with inhibition zones up to 22 mm. The results obtained confirmed the advantages of formulating bioactive silver nanoconjugates and highlight the potential of *S. africana*-derived AgNPs as potent antimicrobial agents against selected bacteria and ABR respiratory pathogens. Further safety and mechanistic studies, including in vivo investigations, are required before biomedical or therapeutic applications can be considered.

## Figures and Tables

**Table 1:** Average crystalline size of silver nanoparticles (SA-AgNPs).

Sample	Average Crystalline Size (nm)
SABM-AG	9
SABE-AG	10
SALM-AG	19
SALA-AG	14
SALD-AG	14

Notes: SABM-AG = bark methanol-AgNPs, SABE-AG = bark ethanol-AgNPs, SALM-AG = leaf methanol-AgNPs, SALA-AG = leaf acetone-AgNPs, SALD-AG = leaf water-AgNPs.

## Data Availability

The original contributions presented in this study are included in the article/supplementary material. Further inquiries can be directed to the corresponding author.

## Supplementary Materials

The following supporting information can be downloaded at: https://www.mdpi.com/article/10.3390/nano16201317/s1, Figure S1: Colour transformation of S. africana extracts (10mg/mL) when mixed with AgNO_3_ (5 mM) solution before (A and C) and after 24 h (B and D) of incubation at room temperature. Figure S2: Colour change of S. Africana Bark-silver nanoparticles (SAB-AgNPs) at various incubation times. Figure S3: Colour change of S. Africana Leaf-silver nanoparticles (SAL-AgNPs) at various incubation times. Figure S4: UV-Vis spectra of methanol extract-based SA-AgNPs, showing the effect of reaction time on nanoparticle synthesis. SA-AgNPs (2.68 mg/mL) were synthesized using AgNO_3_ concentrations of 1, 2, and 5 mM. Figure S5: UV-Vis spectra of leaf ethanol extracts-based AgNPs showing the impact of reaction time. SA-AgNPs (2.68 mg/mL) were synthesized using AgNO_3_ concentrations of 1, 2, and 5 mM. Figure S6: UV-Vis spectra of leaf water extracts-based AgNPs showing the impact of reaction time. SA-AgNPs (2.68 mg/mL) were synthesized using AgNO_3_ concentrations of 1, 2, and 5 mM. Figure S7: The influence of temperature on AgNPs synthesized by S. africana methanol extracts. SA-AgNPs (2.68 mg/mL) were synthesized using AgNO_3_ concentrations of 1, 2, and 5 mM. Figure S8: The influence of temperature on AgNPs synthesized by S. africana ethanol extracts. SA-AgNPs (2.68 mg/mL) were synthesized using AgNO_3_ concentrations of 1, 2, and 5 mM. Figure S9: The influence of temperature on AgNPs synthesized by S. africana water extracts. SA-AgNPs (2.68 mg/mL) were synthesized using AgNO_3_ concentrations of 1, 2, and 5 mM.
