# Supplementary material for: Synthesis, Characterisation, and Antimicrobial Evaluation of Spirostachys africana Extract-Based Silver Nanoparticles Against Selected Bacterial Pathogens, Including Antibiotic-Resistant Strains
Source: Nanomaterials (Basel). 2026 Oct 18;16(20):1317. doi: 10.3390/nano16201317 (PMC13708383; doi:10.3390/nano16201317)
Supplement: Supplementary file 1 [file nanomaterials-16-01317-s001.zip › nanomaterials-4541584-supplementary.pdf]

Supplementary material

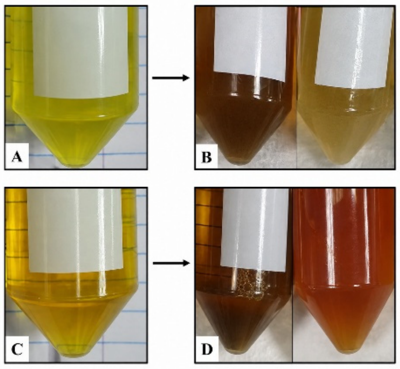

Figure S1: Colour transformation of *S. africana* extracts (10mg/mL) when mixed with $AgNO_3$ (5 mM) solution before (A and C) and after 24 hours (B and D) of incubation at room temperature.

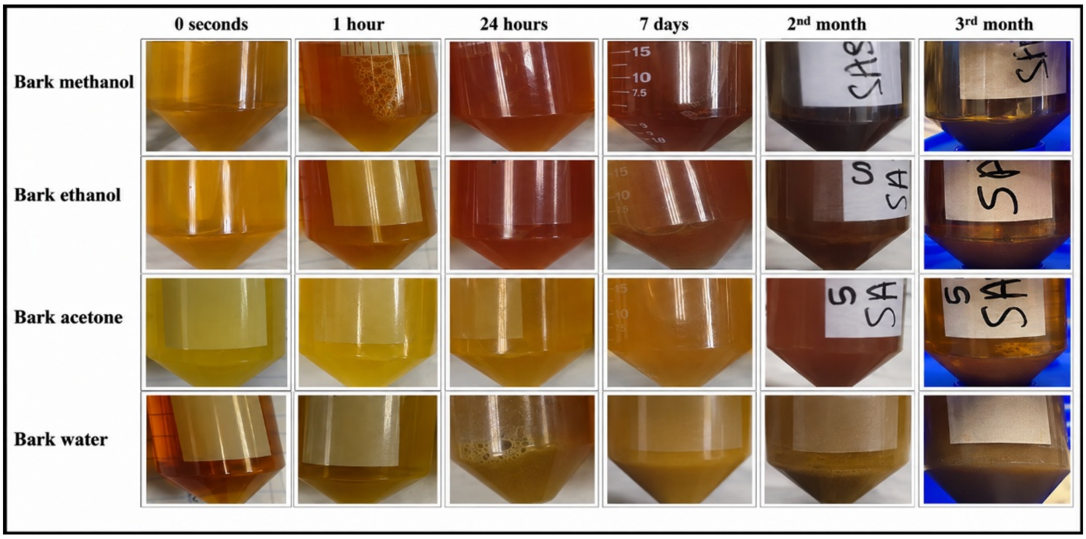

Figure S2: Colour change of *S. Africana* Bark-silver nanoparticles (SAB-AgNPs) at various incubation times.

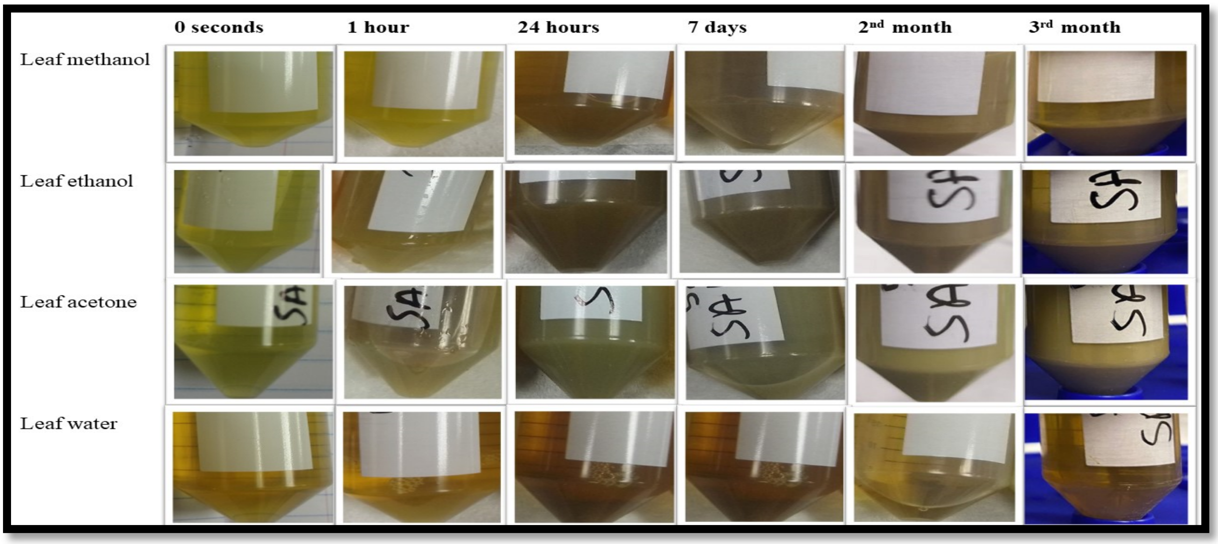

Figure S3: Colour change of *S. Africana* Leaf-silver nanoparticles (SAL-AgNPs) at various incubation times.

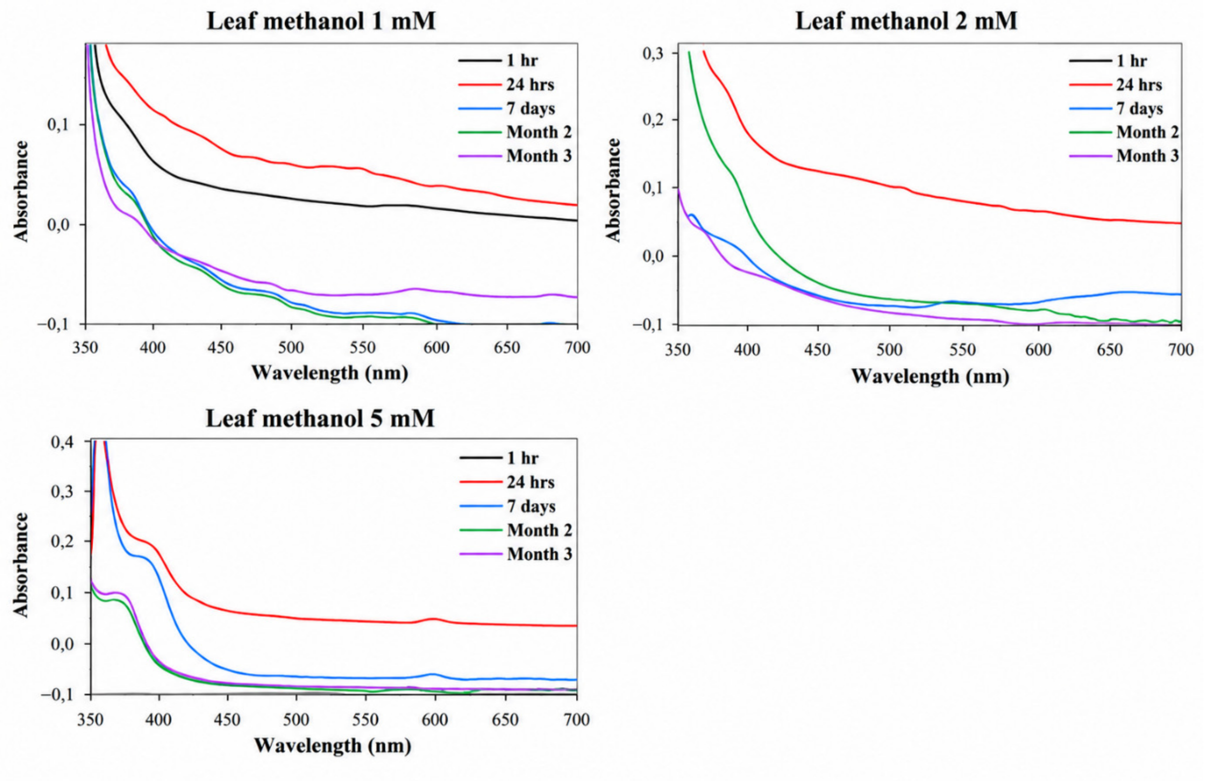

Figure S4: UV-Vis spectra of methanol extract-based SA-AgNPs, showing the effect of reaction time on nanoparticle synthesis. SA-AgNPs (2.68 mg/mL) were synthesized using $AgNO_3$ concentrations of 1, 2, and 5 mM.

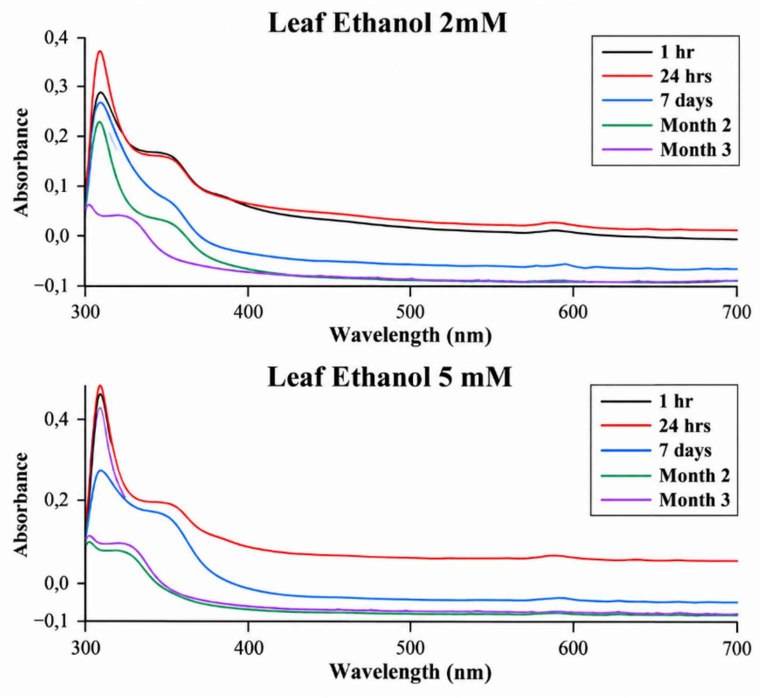

Figure S5: UV-Vis spectra of leaf ethanol extracts-based AgNPs showing the impact of reaction time. SA-AgNPs (2.68 mg/mL) were synthesized using $AgNO_3$ concentrations of 1, 2, and 5 mM.

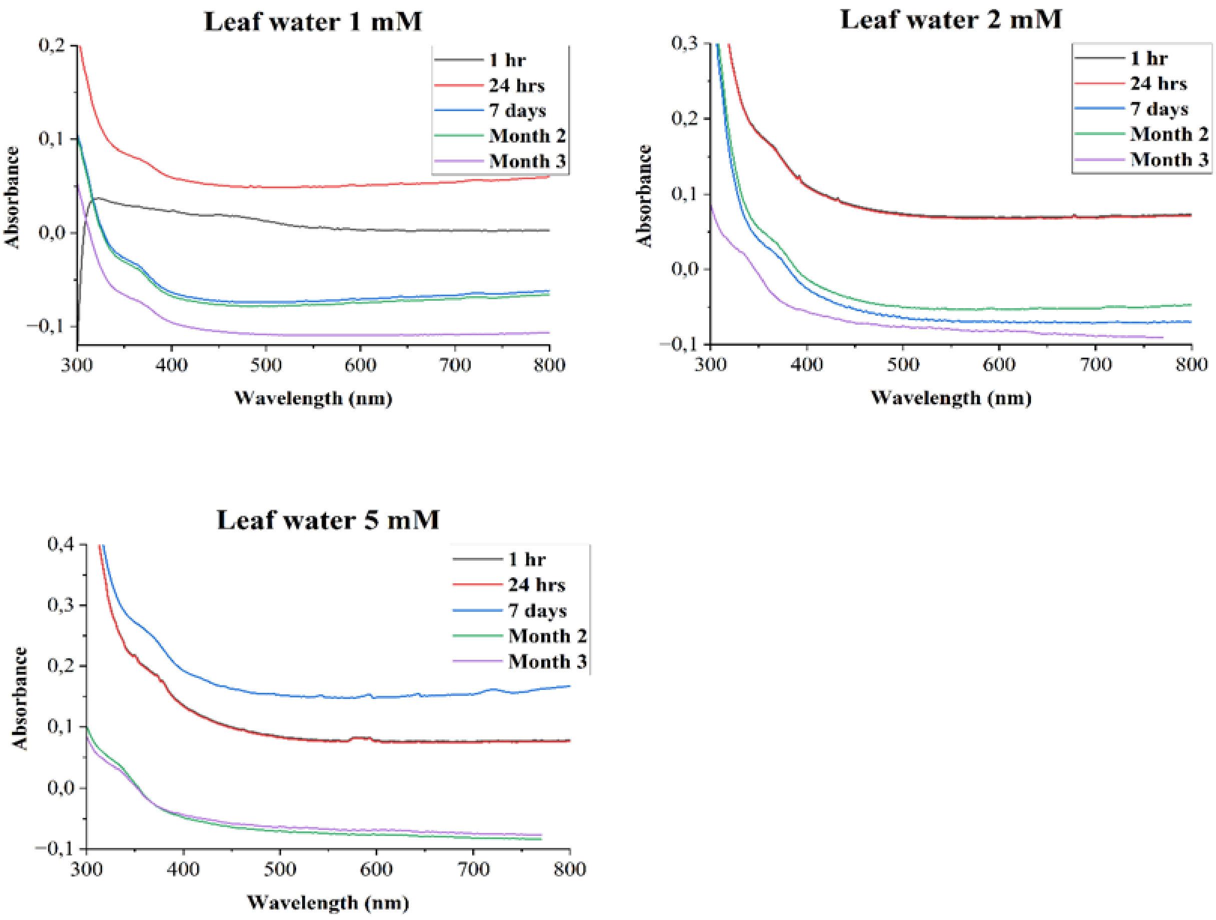

Figure S6: UV-Vis spectra of leaf water extracts-based AgNPs showing the impact of reaction time. SA-AgNPs (2.68 mg/mL) were synthesized using $AgNO_3$ concentrations of 1, 2, and 5 mM.

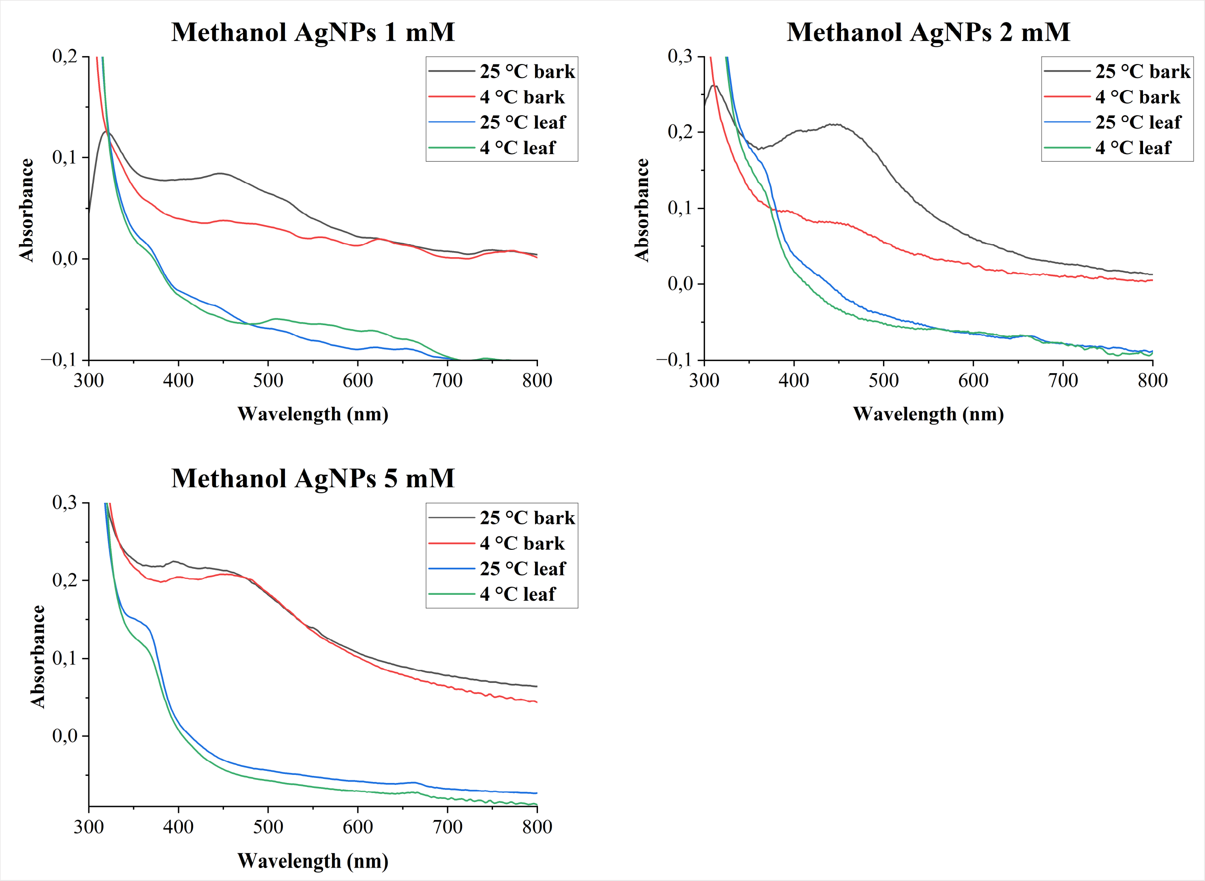

Figure S7: The influence of temperature on AgNPs synthesized by *S. africana* methanol extracts. SA-AgNPs (2.68 mg/mL) were synthesized using $AgNO_3$ concentrations of 1, 2, and 5 mM.

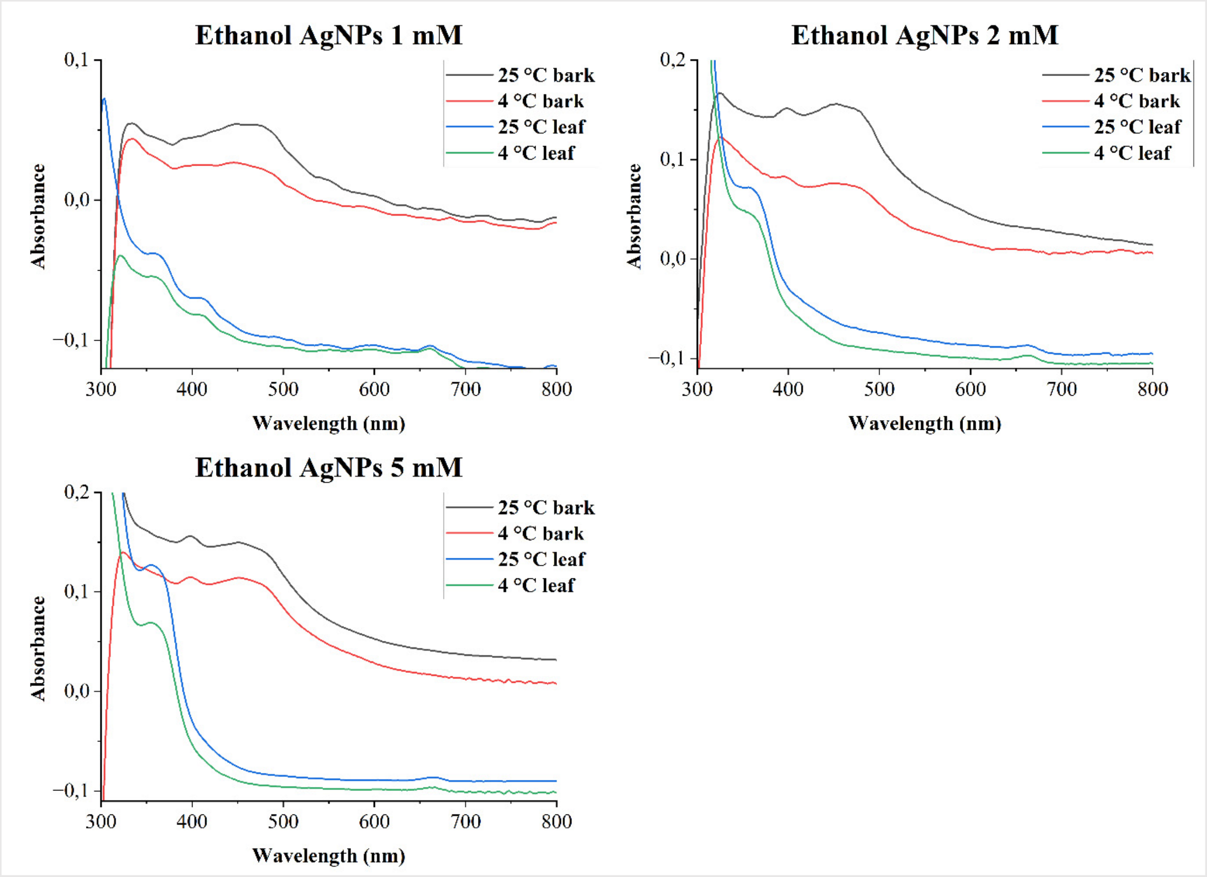

Figure S8: The influence of temperature on AgNPs synthesized by *S. africana* ethanol extracts. SA-AgNPs (2.68 mg/mL) were synthesized using $AgNO_3$ concentrations of 1, 2, and 5 mM.

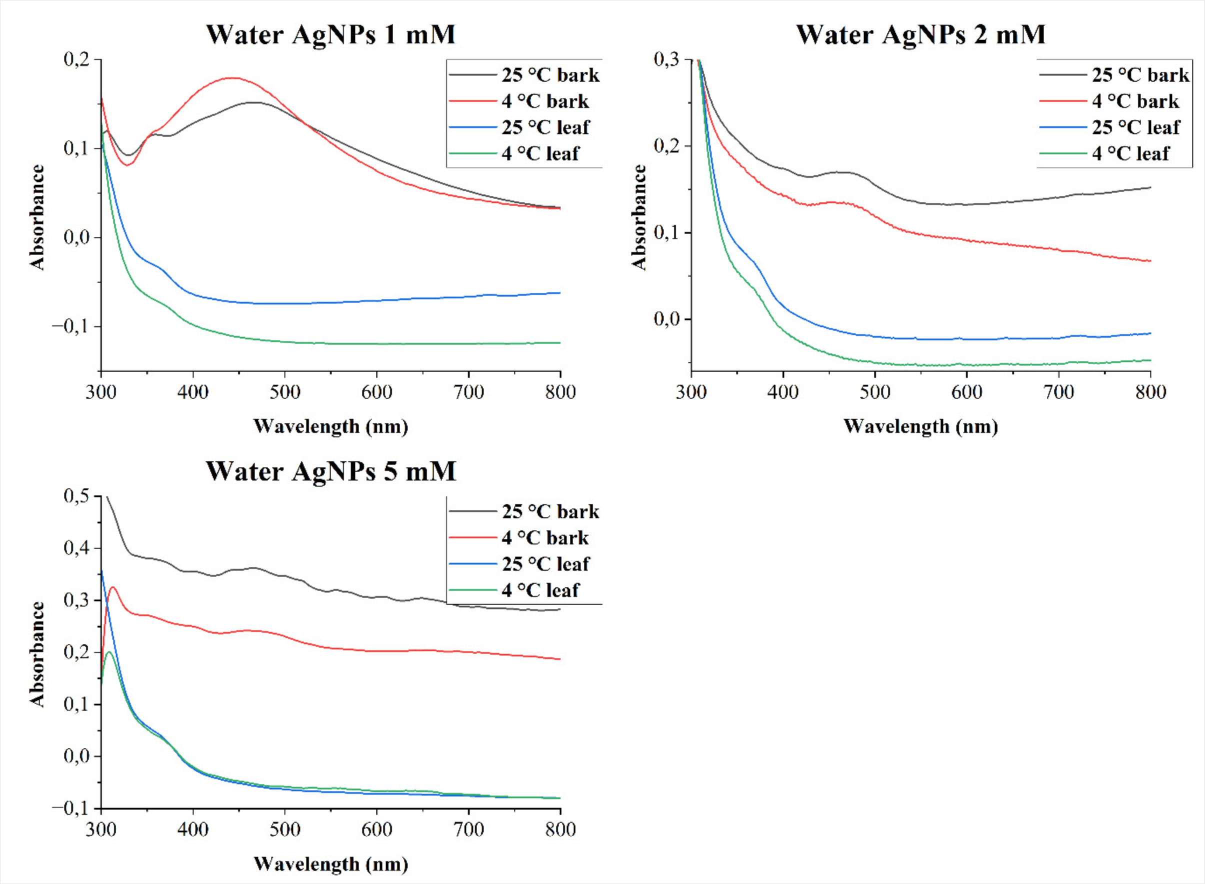

Figure S9: The influence of temperature on AgNPs synthesized by *S. africana* water extracts. SA-AgNPs (2.68 mg/mL) were synthesized using $AgNO_3$ concentrations of 1, 2, and 5 mM.
